# Machine learning-powered antibiotics phenotypic drug discovery

**DOI:** 10.1038/s41598-019-39387-9

**Published:** 2019-03-21

**Authors:** Sannah Zoffmann, Maarten Vercruysse, Fethallah Benmansour, Andreas Maunz, Luise Wolf, Rita Blum Marti, Tobias Heckel, Haiyuan Ding, Hoa Hue Truong, Michael Prummer, Roland Schmucki, Clive S. Mason, Kenneth Bradley, Asha Ivy Jacob, Christian Lerner, Andrea Araujo del Rosario, Mark Burcin, Kurt E. Amrein, Marco Prunotto

**Affiliations:** 1 Roche Pharmaceutical Research and Early Development, Roche Innovation Center Basel, Basel, Switzerland; 2 Roche Pharmaceutical Research and Early Development, Roche Innovation Center Shanghai, Shanghai, China; 3 Discuva Ltd, part of Summit Therapeutics, Merrifield Centre, Cambridge, UK; 4 School of Pharmaceutical Sciences, University of Geneva, Geneva, Switzerland; 5 Present Address: Gilead Sciences, San Francisco, USA; 6 Present Address: NEXUS Personalized Health Technologies, ETH Zürich, and Swiss Institute of Bioinformatics, Zürich, Switzerland; 7 Present Address: I2O Office of Innovation, Roche and Genentech Late Stage Development, Hoffmann-La Roche AG, Basel, Switzerland

## Abstract

Identification of novel antibiotics remains a major challenge for drug discovery. The present study explores use of phenotypic readouts beyond classical antibacterial growth inhibition adopting a combined multiparametric high content screening and genomic approach. Deployment of the semi-automated bacterial phenotypic fingerprint (BPF) profiling platform in conjunction with a machine learning-powered dataset analysis, effectively allowed us to narrow down, compare and predict compound mode of action (MoA). The method identifies weak antibacterial hits allowing full exploitation of low potency hits frequently discovered by routine antibacterial screening. We demonstrate that BPF classification tool can be successfully used to guide chemical structure activity relationship optimization, enabling antibiotic development and that this approach can be fruitfully applied across species. The BPF classification tool could be potentially applied in primary screening, effectively enabling identification of novel antibacterial compound hits and differentiating their MoA, hence widening the known antibacterial chemical space of existing pharmaceutical compound libraries. More generally, beyond the specific objective of the present work, the proposed approach could be profitably applied to a broader range of diseases amenable to phenotypic drug discovery.

## Introduction

Antibiotic drug discovery has been one of the most fascinating tales in the history of modern medicine^1^. This class of drugs still represents, in the consciousness of the general public, the prototypical magic bullet against bacterial pathogens. Over the years, antibiotics have saved more human lives than any other type of drug. Despite the incredible value antibiotics hold for society, the pharmaceutical industry gradually phased out antibiotic research and development due to the virtue of their clinical success. Recently, driven by the rise in antibiotics resistance and the associated public health implications^2^, health regulators, policy-makers and pharma companies have joined forces to incentivize antibiotic R&D^3^.

Despite rapid technological progress, the discovery of novel antibacterial drugs remains challenging. To overcome those difficulties and to move beyond well-known targets, phenotypic drug discovery (PDD) screening methods have been a valid alternative^4^. In a classical phenotypic screening approach, promising antimicrobial compounds are selected on the basis of their empirical ability to prevent cell growth *in vitro*. Results of PDD heavily rely on the chemical diversity of the library used. Though constantly evolving and enriching over time, the compound collections designed for drug development are biased for chemical features optimized to increase permeability in eukaryotic cells, routine targets of pharmaceutical research. Prokaryotic specific structures, *e.g*., bacterial cell walls, and specific mechanisms, *e.g*., efflux pumps, degrading enzymes or biofilm formation, reduce even further the spectrum of useful compounds capable of inducing bacteria cell death or preventing bacteria cell growth. Corporate compound libraries have therefore a limited chemical diversity^5^ in relation to bacteria drugability^6^.

The present study investigates compounds from such libraries, which display weak potency of inducing bacteria cell death or inhibiting bacteria growth under general screening conditions, searching for compound-induced phenotypic modulation of individual cellular features to identify potential candidates for development into antibiotics. We investigated this *grey* chemical matter (defined as compounds capable of inducing phenotypic modulation but only weakly active in inducing bacteria cell death or inhibiting bacteria growth at typical screening concentration) adopting a combined multiparametric high content screening (HCS) approach using a combination of PDD methods and a robust data analysis pipeline powered by machine learning (ML). The present proof of concept study showed that this approach allows identification of compounds with novel mode of action (MoA) amenable to medicinal chemistry evolution into leads.

By sharing our methodology and the related initial results, we do hope to encourage other research teams to explore their own compound collections using this alternative paradigm in order to identify novel antibiotics.

## Results

### The chemical space of the Roche pharma library is limited in respect to antibacterial-susceptibility

**Figure 1:**
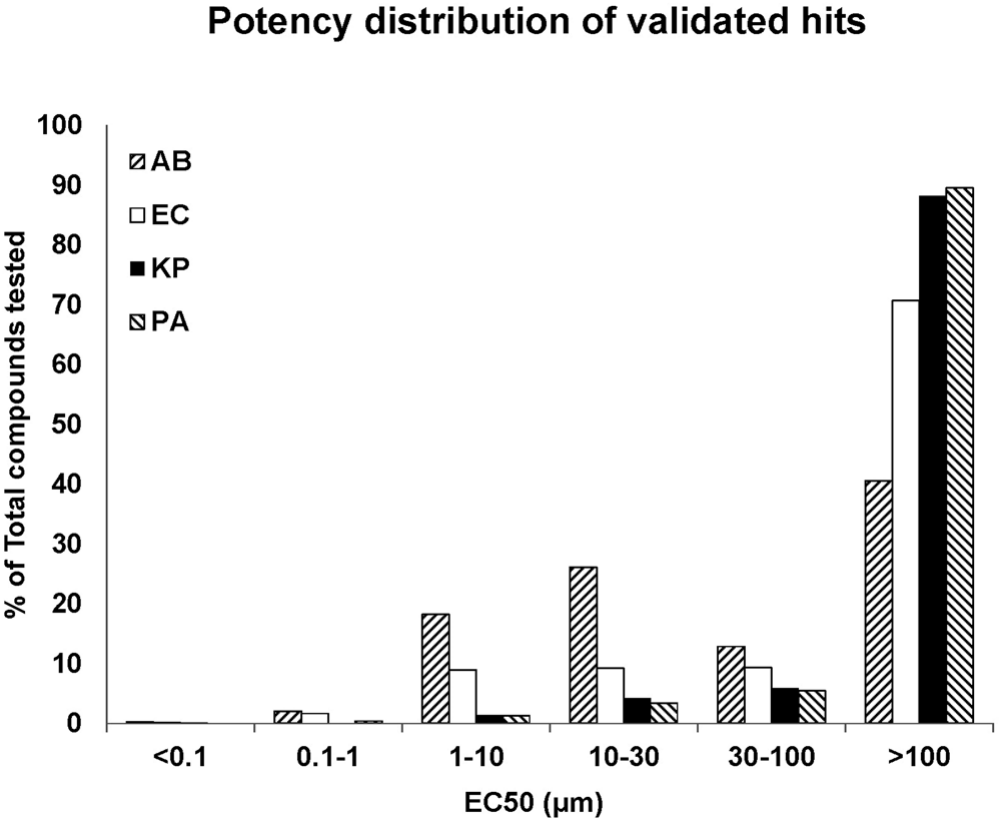
Antibacterial susceptibility of prioritized hits extracted from the Roche/Genentech compound collection. The histogram shows the potency distribution of 750 compounds active against one or more key Gram negative bacterial species. AB: *A. baumannii*, EC: *E. coli*, KP: *K. pneumoniae*, PA: *P. aeruginosa*, EC50: effective compound concentration to inhibit growth by 50%.

The antibacterial activity of 1.5 million compounds from the Roche compound library were tested at a single concentration (40 μM) against 4 Gram-negative (GN) pathogens: i) *Acinetobacter baumannii* (ATCC 17978), ii) *Escherichia coli* (BW25113), iii) *Klebsiella pneumoniae* (NCTC 13438) and iv) *Pseudomonas aeruginosa* (NCTC 11451). To measure inhibition the reduction in bacterial growth (OD 600 nm; relative to growth in the absence of compound) was determined 16 hrs after compound addition (data not shown). Initially approximately 10’000 compounds were identified that inhibited growth more than 50% of any one strain tested. Among the hits were many compounds from historic antibiotics projects. After removing known antibiotics and frequent hitters, the remaining compounds were prioritized based on novelty, potency, chemical structure, and availability of purified powder material. In total 750 hits were validated in a subsequent 10-point dose-response screen (EC50) against the pathogens used in the initial screen. Figure 1 displays an overview of the results after grouping the data into 6 different potency categories. Only a limited fraction of the tested compounds (0.05%) displayed potent (<0.1 μM EC50) antibacterial activity. Clear differences in susceptibility are observed amongst the 4 GN species especially in the EC50 intervals between 1 and 10 µM and 10 and 30 µM. Wild type *A. baumannii* is most susceptible to active compounds whereas fewer compounds were found active against wild-type *E. coli*. *E. coli*-susceptibility is however still significantly higher than those for *K. pneumoniae* or *P. aeruginosa*.

### Compound-induced phenotypic changes results in a specific bacterial phenotypic fingerprint defined at the lowest effective dose

In order to move beyond bacteria cell growth inhibition as phenotypic screening readout we explored alternative indicators for compound activity at lower concentrations and/or earlier time points. Treatment with antibiotics is known to induce changes to a range of individual cell features which can be captured with microscopy^7^. In our approach we visualize these changes with fluorescent stains for membrane, DNA and membrane permeability and use HCS automated microscope (Perkin-Elmer Opera QEHS) and instrument integrated software (Acapella) to capture and quantify the changes to individual features. The major part of the features relates to cell morphology, fluorescence intensity and (sub)cellular distribution, and together the unique pattern in change constitute the compound induced phenotype or bacterial phenotypic fingerprint. A full list with description of the derived parameters can be found in Supplementary Table S1.

**Figure 2:**
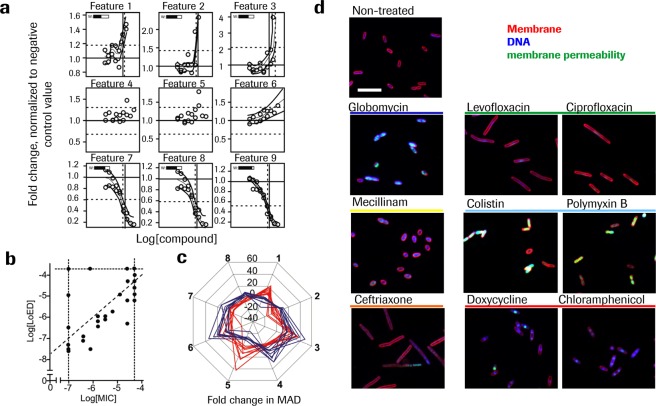
Bacteria phenotypic modulation is defined by a lowest effective dose. (**a**) Schematic illustration of LOED calculation method: Dose-dependent response curves for a small subset of nine features selected to represent different types of response profiles are shown to illustrate the process: LOED is indicated as solid vertical bar at the same location in all the panels, and calculated in a consensus approach as the weighted mean of all detected individual LOEDs (dashed vertical lines). Features 1–3 and 7–9 reaches respectively positive – or negative deflection points and individual LOEDs can be derived, while features 4–6 do not contribute, either due to missing curve fit (4&5) or no significant deflection (6). The weights of the contributing features are defined as the goodness of the respective curve fit (variable “w”, top left in each panel). (**b**) Correlation between MIC and LOED for *E. coli* WT. Horizontal and vertical stabled lines indicate concentration range tested. Similar value for LOED and MIC is indicated with the diagonal line. Panel (**c**) Comparison of the antibiotic treatment induced changes to eight selected features quantified with HCS and expressed as fold MAD of non-treated control samples, for two compounds with similar LOED, doxycycline (red) and globomycin (blue). For detailed feature description see Supplementary Table S1 d) *E. coli* treated with the indicated compound for 1h with a concentration at 4x LOED. Images of the fluorescent stains were acquired with the Opera QEHS reader, 60x magnification. Scale bar corresponds to 10 μm.

Next, we set out to identify a robust definition of the lowest compound concentration capable of inducing significant changes to the bacterial phenotype. Spline fits were generated for all individual HCS derived features. For each feature the concentration where a significant change is reached was defined as the spline fit point where the complete confidence interval exceeded 3 times median absolute deviation (MAD) of untreated samples from median values of control samples. A set of 9 features representative of different response profiles derived from the same compound dose response curve are shown in Fig. 2a to illustrate the logic of LOED determination. Features 1–3 and 7–9 all contribute, reaching respectively the upwards or downward deflection point, while features 4–6 do not contribute, because of missing curve fit or no significant deflection within the concentration range. Consensus *lowest effective dose* (LOED) was defined as the composite of all feature values with significant change by taking the mean of the individual values weighted on the goodness of the fit. No LOED was defined in case of less than 3 individual features being significant. See also Fig. S1 for details. This approach resulted in a finely tuned, statistically-stable characterization of the lowest effective, sub-lethal dose inducing bacterial phenotypic modulation, as indicated by the generally low standard deviation of the LOED for individual compounds between independent experiments (Tables 1, 2 and -S2).

**Table 1:** Similarity score for known antibacterial compounds on *E. coli ΔTolC*. Similarity score for compound effect on *E. coli ΔTolC* derived with Random Forest analysis from compiled phenotypic changes quantified across multiple experiments. The Random Forrest model is established using the parameter set for the six reference antibiotics and non-treated samples using the values at 2 and 4 fold LOED. Each analysis combines values from 2-3 experiments with n = 1-3. The number of data points for each reference condition is reduced to a similar number by automated selection of data points for the individual experiments. The similarity score in the individual experiment is the frequency of matching prediction expressed as a fraction of 1, where a higher value represents a higher similarity. Fingerprint quality: “Clear”: At least one parameter with >10 fold change in >85% of the individual datapoints at 4x LOED. “Weak”, no single parameter with consistent change required for classification as “clear”, but visual inspection reveals presence of a systematic weaker fingerprint including >5 parameters. LOED is derived for each individual experiment with n = 1-3.

Test Compound with known MoA/target	Fingerprint quality		Set of reference treatments for random forest model generation							Number of independent analysis	Log[LOED] Median ± Stdev (N)
			Ceftriaxone	Colistin	Doxycycline	Globomycin	Levofloxacin	Mecillinam	DMSO		
Ceftriaxone *Cell-wall*	clear	out of bag analysis	**0.92** ± *0.03*	**0.02** ± *0.03*	**0.01** ± *0.01*	**0.01** ± *0.00*	**0.04** ± *0.01*	**0.00** ± *0.02*	**0.01** ± *0.01*	11	**−7.3 ± **0.2 (25)
Colistin *Membrane integrity*	clear		**0.02** ± *0.01*	**0.90** ± *0.01*	**0.01** ± *0.01*	**0.03** ± *0.01*	**0.01** ± *0.01*	**0.01** ± *0.02*	**0.02** ± *0.01*		**−6.2 ± **0.3 (25)
Doxycycline *Ribosome*	clear		**0.01** ± *0.01*	**0.01** ± *0.01*	**0.92** ± *0.02*	**0.01** ± *0.01*	**0.03** ± *0.01*	**0.01** ± *0.02*	**0.02** ± *0.01*		**−6.2 ± **0.2 (25)
Globomycin *Lipoprotein export*	clear		**0.01** ± *0.00*	**0.03** ± *0.00*	**0.01** ± *0.01*	**0.91** ± *0.05*	**0.01** ± *0.01*	**0.03** ± *0.01*	**0.01** ± *0.02*		**−6.4 ± **0.6 (23)
Levofloxacine *Topoisomerase*	clear		**0.04** ± *0.01*	**0.01** ± *0.01*	**0.03** ± *0.01*	**0.01** ± *0.00*	**0.89** ± *0.03*	**0.01** ± *0.00*	**0.01** ± *0.00*		**−7.9 ± **0.6 (25)
Mecillinam *Cell-wall*	clear		**0.00** ± *0.00*	**0.01** ± *0.00*	**0.01** ± *0.01*	**0.02** ± *0.01*	**0.00** ± *0.00*	**0.92** ± *0.02*	**0.03** ± *0.01*		**−6.4 ± **0.3 (25)
DMSO			**0.01** ± *0.01*	**0.03** ± *0.01*	**0.03** ± *0.01*	**0.01** ± *0.04*	**0.02** ± *0.01*	**0.06** ± *0.03*	**0.83** ± *0.06*		
Cefepim *Cell-wall*	clear	Similarity score towards reference antibiotics	**0.80** ± *0.00*	**0.05** ± *0.00*	**0.02** ± *0.00*	**0.02** ± *0.00*	**0.08** ± *0.00*	**0.01** ± *0.00*	**0.02** ± *0.00*	2	**−7.8 ± **0.2 (4)
Polymyxin B *Membrane integrity*	clear		**0.05** ± *0.01*	**0.75** ± *0.04*	**0.04** ± *0.01*	**0.08** ± *0.03*	**0.03** ± *0.01*	**0.01** ± *0.00*	**0.03** ± *0.01*	5	**−5.8 ± **0.5 (10)
Chloramphenicol *Ribosome*	clear		**0.01** ± *0.01*	**0.02** ± *0.01*	**0.75** ± *0.06*	**0.03** ± *0.01*	**0.04** ± *0.03*	**0.06** ± *0.02*	**0.06** ± *0.02*	5	**−5.5 ± ** *0.2 (9)*
Clarithromycin *Ribosome*	clear		**0.01** ± *0.01*	**0.02** ± *0.01*	**0.77** ± *0.04*	**0.02** ± *0.01*	**0.04** ± *0.02*	**0.06** ± *0.01*	**0.08** ± *0.01*	6	**−5.5 ± **0.1 *(10)*
AZ-LolCDE *Lipoprotein export*	clear		**0.01** ± *0.00*	**0.07** ± *0.01*	**0.01** ± *0.00*	**0.79** ± *0.04*	**0.01** ± *0.00*	**0.09** ± *0.04*	**0.01** ± *0.00*	3	**−6.7 ± ** *0.3 (5)*
Ciprofloxacin *Topoisomerase*	clear		**0.17** ± *0.05*	**0.03** ± *0.01*	**0.04** ± *0.01*	**0.04** ± *0.01*	**0.68** ± *0.07*	**0.01** ± *0.00*	**0.02** ± *0.01*	3	**−7.7 ± ** *0.2 (7)*
Norfloxacine *Topoisomerase*	clear		**0.11** ± *0.02*	**0.03** ± *0.01*	**0.06** ± *0.01*	**0.03** ± *0.02*	**0.74** ± *0.04*	**0.01** ± *0.00*	**0.02** ± *0.01*	2	**−7.4 ± ** *0.2 (4)*
Avibactam *β-lactamase and cell-wall*	clear		**0.01** ± *0.00*	**0.02** ± *0.00*	**0.02** ± *0.02*	**0.04** ± *0.01*	**0.01** ± *0.00*	**0.74** ± *0.07*	**0.16** ± *0.05*	2	**−4.9 ± ** *0.2 (4)*
CCCP *Membrane potential*	bi-phasic		*NA*	*NA*	*NA*	*NA*	*NA*	*NA*	*NA*		*NA*
GyrB/ParE ATPase *Gyrase B*	clear		**0.03** ± *0.03*	**0.04** ± *0.03*	**0.36** ± *0.14*	**0.05** ± *0.02*	**0.43** ± *0.15*	**0.03** ± *0.02*	**0.05** ± *0.03*	8	**−7.4 ± ** *0.3 (11)*
MD3 *SPase1*	weak		**0.01** ± *0.01*	**0.04** ± *0.05*	**0.11** ± *0.08*	**0.05** ± *0.05*	**0.03** ± *0.01*	**0.14** ± *0.09*	**0.42** ± *0.14*	1	**−4.3 ± ** *0.6 (3)*
Nitrofurantoin *Unknown*	clear		**0.15** ± *0.12*	**0.11** ± *0.04*	**0.13** ± *0.08*	**0.10** ± *0.11*	**0.33** ± *0.04*	**0.03** ± *0.04*	**0.10** ± *0.03*	3	**−5.2 ± ** *0.2 (7)*
Nitroxoline *Unknown*	no		*NA*	*NA*	*NA*	*NA*	*NA*	*NA*	*NA*		*NA*
Triclosan *FabI*	clear		**0.17** ± *0.11*	**0.28** ± *0.07*	**0.08** ± *0.02*	**0.15** ± *0.07*	**0.07** ± *0.05*	**0.09** ± *0.03*	**0.16** ± *0.05*	5	**−5.8 ± ** *0.7 (11)*
Trimethoprim *DNA synthesis*	clear		**0.08** ± *0.05*	**0.06** ± *0.03*	**0.24** ± *0.11*	**0.14** ± *0.11*	**0.23** ± *0.11*	**0.08** ± *0.03*	**0.16** ± *0.03*	2	**−6.9 ± ***0.3 (*7)

**Table 2:** Identification of MoA for new antibacterial compound classes. Similarity score for compound effect on *E. coli ΔTolC* derived with Random Forest analysis from compiled phenotypic changes quantified across multiple experiments to assess the MOA for the five active test compounds from compound class 1. Median value of classification in 2–5 experiment series, each 2 independent experiments. Only the median is displayed for clarity, for more details see Table-S3. Column 3–9, standard set of reference compounds used to build the random forest model. Each of the following columns is for the similarity score generated with a slightly different reference set where the indicated test compound has replaced the compound with the closest profile in the reference set. Only the similarity score towards the new reference compound is displayed.

	Default *E. coli* reference compounds								Custom reference compounds										Log[LOED] Median +/- stdev (N)
		Ceftriaxone	Colistin	Doxycycline	Globomycin	Levofloxacin	Mecillinam	DMSO	GyrB/ParE- ATPase	MD3	Nitrofurantoin	Triclosan	Trimethoprim	1a	1b	1h	1j	1o	
Test compounds	Ceftriaxone	**0.90**	0.02	0.01	0.01	0.05	0.01	0.01	0.04	0.00	0.07	0.03	0.04	0.02	0.03	0.02	0.00	0.01	−7.3 ± 0.3 (15)
	Colistin	0.02	**0.90**	0.01	0.03	0.01	0.01	0.02	0.02	0.01	0.04	0.04	0.03	0.04	0.04	0.04	0.01	0.01	−6.2 ± 0.3 (15)
	Doxycycline	0.01	0.01	**0.92**	0.00	0.03	0.01	0.02	0.17	0.02	0.08	0.01	0.07	0.01	0.01	0.01	0.03	0.03	−6.2 ± 0.3 (15)
	Globomycin	0.01	0.01	0.01	**0.95**	0.01	0.02	0.00	0.01	0.01	0.02	0.22	0.06	0.22	0.18	0.21	0.02	0.01	−6.6 ± 0.4 (14)
	Levofloxacin	0.05	0.02	0.03	0.01	**0.88**	0.01	0.01	0.24	0.00	0.38	0.01	0.14	0.01	0.01	0.01	0.00	0.00	−7.9 ± 0.7 (15)
	Mecillinam	0.01	0.01	0.01	0.02	0.00	**0.92**	0.03	0.02	0.01	0.02	0.01	0.03	0.01	0.01	0.01	0.03	0.03	−6.7 ± 0.4 (15)
	DMSO	0.02	0.03	0.04	0.01	0.02	0.04	**0.83**	0.02	0.07	0.05	0.02	0.05	0.03	0.01	0.02	0.07	0.08	
	GyrB/ParE ATPase	0.04	0.03	**0.63**	0.01	0.23	0.02	0.03	**0.90**	0.02	0.30	0.03	0.27	0.04	0.02	0.00	0.03	0.02	−7.4 ± 0.0 (2)
	MD3	0.01	0.03	0.13	0.01	0.02	0.17	0.59	0.06	**0.92**	0.04	0.03	0.09	0.05	0.02	0.03	**0.71**	**0.63**	−4.3 ± 0.6 (11)
	Nitrofurantoin	0.20	0.12	0.19	0.04	0.31	0.05	0.09	0.25	0.01	**0.88**	0.05	0.34	0.05	0.07	0.05	0.02	0.02	−5.2 ± 0.2 (7)
	Triclosan	0.17	0.28	0.08	0.15	0.07	0.09	0.18	0.03	0.04	0.14	**0.92**	0.04	**0.67**	**0.64**	**0.69**	0.02	0.05	−5.8 ± 0.7 (11)
	Trimethoprim	0.12	0.07	0.36	0.06	0.18	0.10	0.12	0.35	0.04	0.49	0.04	**0.89**	0.04	0.04	0.03	0.04	0.03	−6.9 ± 0.5 (4)
	1a	0.27	0.26	0.07	0.21	0.08	0.06	0.16	0.05	0.02	0.14	**0.71**	0.06	**0.92**	**0.79**	**0.82**	0.02	0.03	−5.5 ± 0.4 (11)
	1b	0.30	0.24	0.07	0.18	0.08	0.05	0.09	0.04	0.02	0.14	**0.72**	0.05	**0.69**	**0.92**	**0.74**	0.02	0.02	−4.9 ± 0.4 (11)
	1 h	0.29	0.25	0.07	0.19	0.07	0.06	0.13	0.03	0.02	0.15	**0.73**	0.04	**0.80**	**0.75**	**0.92**	0.01	0.03	−5.2 ± 0.2 (11)
	1j	0.02	0.02	0.08	0.01	0.01	0.36	0.45	0.08	**0.77**	0.06	0.03	0.10	0.05	0.02	0.04	**0.92**	**0.75**	−4.3 ± 0.1 (6)
	1o	0.01	0.03	0.17	0.01	0.02	0.23	0.51	0.08	**0.74**	0.05	0.04	0.09	0.05	0.03	0.03	**0.87**	**0.91**	−4.3 ± 0.2 (11)

Next we assessed the relationship between the minimal inhibitory concentration (MIC), which is the lowest concentration of antibiotic that will inhibit bacterial growth, and the LOED for a set of test compounds. The LOED proved to be lower than the MIC with a median MIC/LOED ratio of 3.9 for *E. coli* wild type (Fig. 2b). We further explored the significance of compounds having similar LOED levels examining their similarity at single feature levels. Doxycycline and Globomycin, respectively inhibiting protein synthesis and lipoprotein export, have LOEDs in the same range (log[LOED]: −5.73 ± 0.68 and −5.20 ± 0.66 respectively) and induce clearly different phenotypes, illustrated by differences in the effect on selected features (Fig. 2c). However, compounds with similar MoA irrespective of their individual LOED value induce visibly similar changes to the bacteria morphology as seen in pictures extracted at 4x LOED supporting clustering according to similarity of phenotypic fingerprints (Fig. 2d).

### ML algorithms capture the complexity of bacterial phenotypic fingerprints

**Figure 3:**
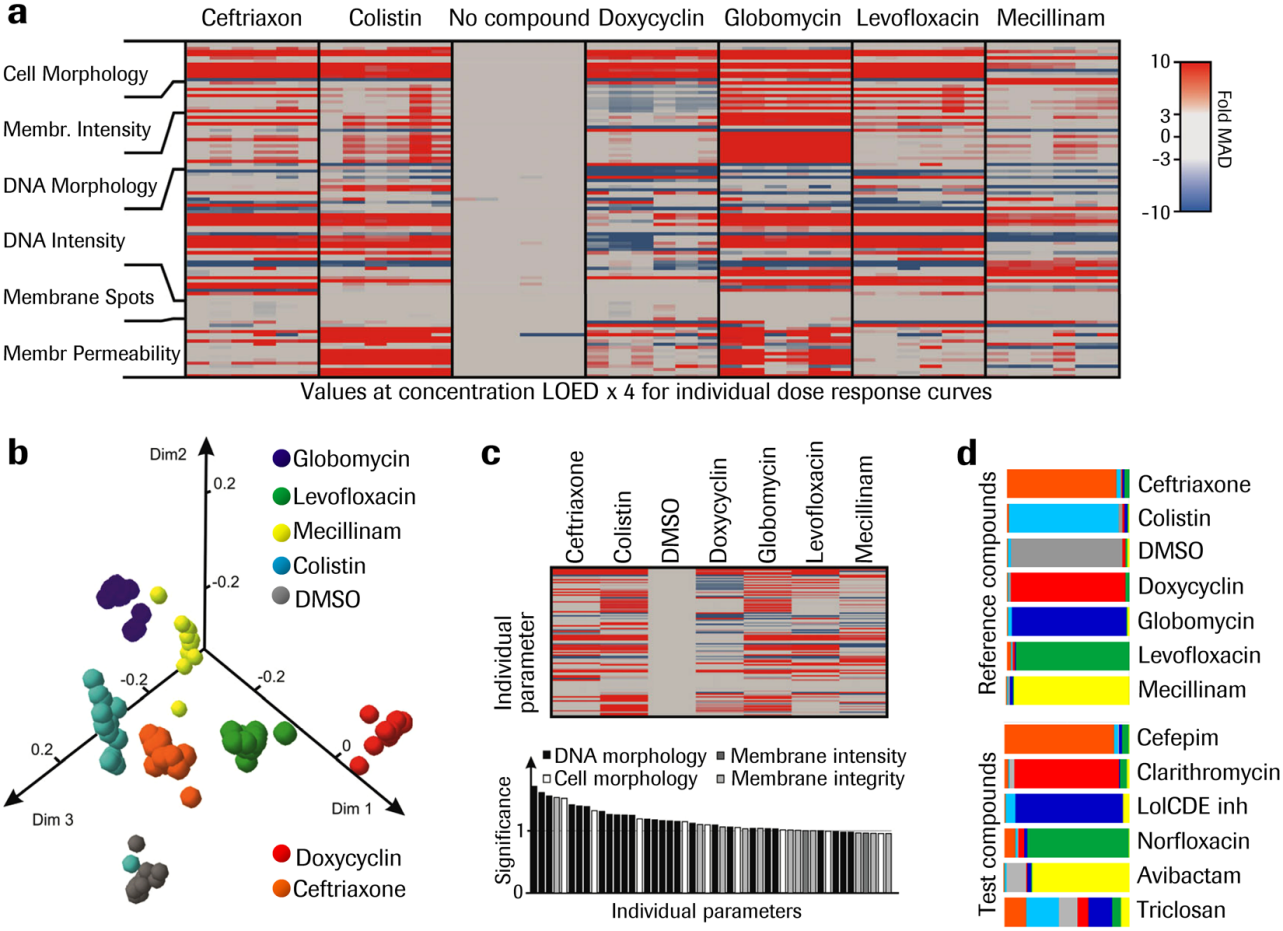
ML algorithms handle bacteria phenotypic fingerprint complexity. (**a**) Compiled changes to individual features, each a change to cell morphology and/or fluorescence stain intensity captured with HCS at 4x LOED induced by a set of reference compounds for *E. coli ΔTolC*. Each column corresponds to the data from one well and the rows to individual features. The color indicates the change in the feature value, expressed as fold MAD over median calculated for all non-treated samples. For each compound, results from three experiments are shown, with n = 2-3. (**b**) Corresponding similarity-based projection of the random forest distance matrix into 3 dimensions using multidimensional scaling, showing clear separation between data points belonging to different compounds and close proximity of those belonging to the same compounds, forming clusters for the combined data points at 2x- and 4xLOED. The number of data points are reduced by down sampling to be equal for all reference conditions. (**c**) Upper panel: Archetypes for the set of reference antibiotics. The value calculation and heatmap scaling is the same as in panel a. Lower panel: distribution of feature importance contributing to archetypes. (**d**) Out-of-bag validation of the random forest classification model for reference compound set (upper panel) or similarity score of test compounds (lower panel). The similarity score in the individual experiment is the frequency of matching prediction expressed as a fraction of 1, where a higher value represents a higher similarity.

To analyze the compiled morphological changes of test compounds induced at 2–4x LOED we used a set of reference antibiotics with known MoA. A unique morphology signature or fingerprint was generated for each compound by using the results from several experiments revealing sets of features that consistently change across experiments (Fig. 3a). These results were analyzed using a random forest model, which is a ML algorithm, to assess the similarity of multiparametric phenotypes to that of the reference antibiotic set. The similarity-based projection of the random forest distance matrix into 3 dimensions shows a clear separation between data points belonging to compounds with a different MoA and an equally clear clustering of data points belonging to compounds with a similar MoA (Fig. 3b). For the set of reference antibiotics, the data points closest to the cluster centers identify prototypical phenotypes or so called *archetypes* (Fig. 3c, upper panel). To create clearly defined archetypes, multiple parameters are necessary to separate data points belonging to different compounds as shown by the flat distribution of feature importance (Fig. 3c, lower panel). At the end of the training phase, an out-of-bag validation of the random forest classification model showed a successful separation and correct classification of the reference compounds set, illustrated by the width of bar segments that indicate the distribution of classification (Fig. 3d, upper panel). The trained model was applied to predict the similarity distribution of test compounds relative to the reference set, visualized by the segmented bars. The test compounds with known MoA matched the corresponding reference compounds with a similarity score well beyond 60%, while compounds with different MoA than the reference componds stayed below 40%. For example, Ciprofloxacin shows a similarity score of 68% towards Levofloxacin and Polymyxin B 75% towards Colistin (Fig. 3d, lower panel). Interestingly, Avibactam had a 74% similarity score towards Mecillinam, strongly supporting the hypothesis^8^ that in addition to inhibition of beta-lactamase, it also exerts an antibacterial activity through inhibition of the penicillin binding protein 2.

### Bacterial phenotypic fingerprints quantitatively identify compounds with similar MoA

The performance of the ML-powered HCS approach was systematically assessed across multiple experiments both on *E. coli* WT and the *ΔTolC* mutant to predict the MoA of test compounds. A high similarity score was observed for all compounds compared to the reference antibiotic with matching MoA (range of 0.6–0.8, Table 1). For example, the ribosomal inhibitors Clarithromycin and Chloramphenicol showed the highest similarity score to Doxycycline, respectively 0.72 and 0.78. Compounds with a known MoA different from the selected reference compounds displayed a much lower similarity score (<0.5). Few reference antibiotics showed no clear change in phenotype even though the bacterial cell count was reduced, *e.g*., Nitroxoline. The similarity score for the same set of reference compounds in individual experiments proved to be highly reproducible and similar in screening either *E. coli* wild type or *ΔTolC* mutants (Supplemental Table 1).

### Bacterial phenotypic fingerprints of novel compounds can guide the SAR

We then sought to demonstrate that phenotypic fingerprints can be used to both identify a likely target and guide the structure activity relation (SAR) analysis of a chemical series.

**Figure 4:**
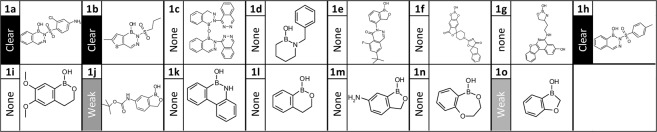
Identification of MoA for new antibacterial compound classes. Structures of the compound series 1. Indicated left of the structure is the quality of the fingerprint. Fingerprint quality: “Clear”: At least one parameter with >10 fold change in >85% of the individual datapoints at 4-fold LOED. “Weak”, no single parameter with consistent change required for classification as “clear”, but visual inspection reveals presence of a systematic weaker fingerprint including >5 parameters.

To do this we selected a set of compounds both closely and more distantly related to a boronate compound series (Table 2 and Fig. 4), which has previously been described to most likely target FabI. Out of the 15 tested boronate analogues, 5 displayed a phenotypic fingerprint. However none of these analogs showed a high similarity score toward the standard set of reference antibiotics. Hence, we modified the reference set by systematically replacing one compound with other reference compounds representing different MoA (GyrB/ParE ATPase inhibitor, Triclosan, Trimethoprim, MD3, and Nitrofurantoin) as well as the test compounds. A high similarity score (ranging from 0.66–0.72) was observed for 3 of the 5 antibacterial compounds (**1a**, **1b** and **1h**) to Triclosan, which is a known FabI inhibitor. Interestingly, the 3 compounds share a common 2-sulfonylated diazaborinines structural motif which is absent in the other 12 selected analogues. The two other active compounds, *i.e*. **1j** and **1o**, induced a weak but distinct phenotype close to the highest tested concentration at 200 µM, which surprisingly  clustered towards each other and the extra reference antibiotic MD3, which is a known signal peptidase 1 inhibitor, rather than Triclosan. Those results suggest that the ML-powered HCS tool can systematically scan similarities toward reference compounds with known MoA identifying key SAR motifs.

### Bacterial phenotypic fingerprint can be complemented by molecular information

**Figure 5:**
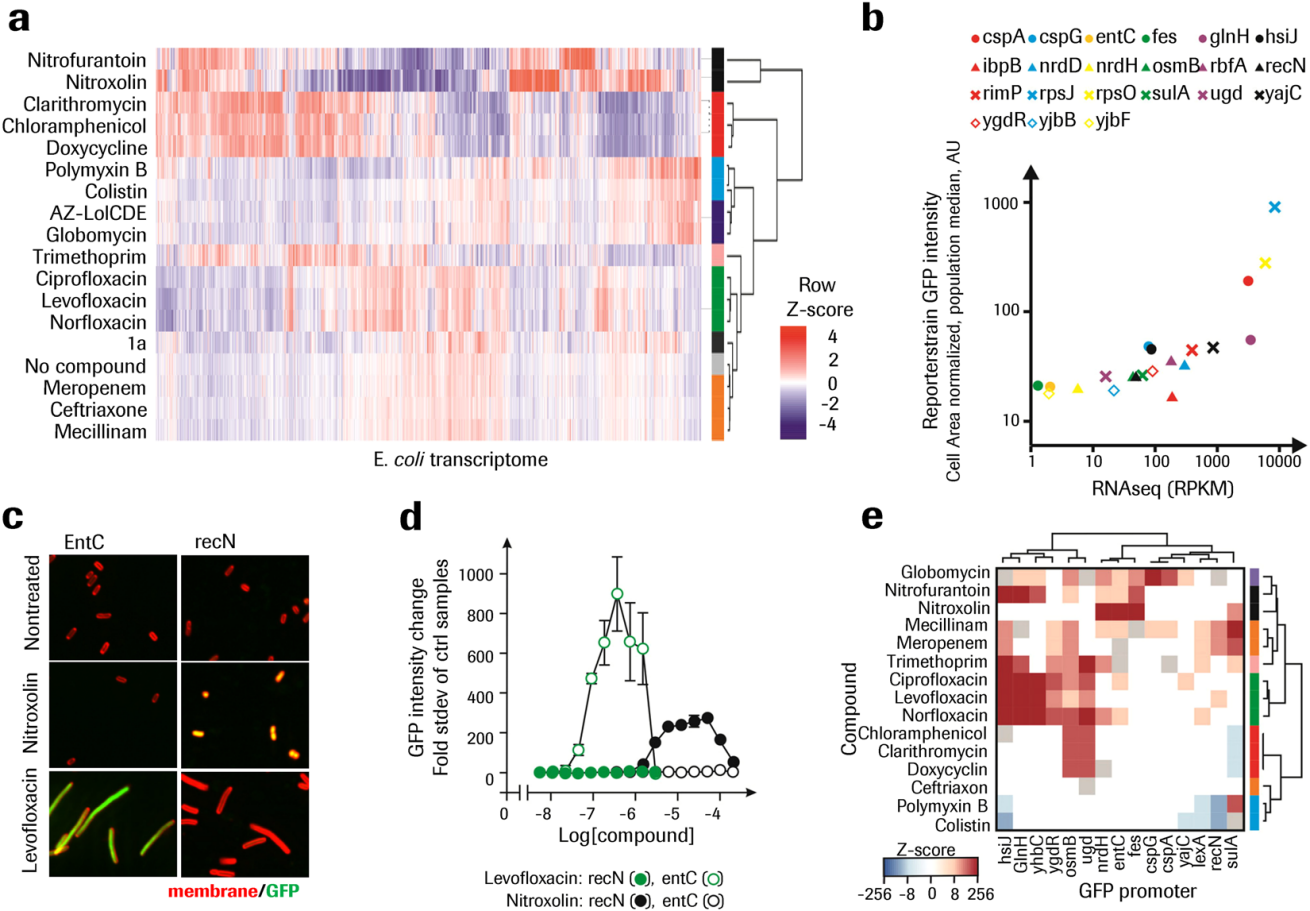
Bacteria phenotypic fingerprint can be powered by molecular information. (**a**) Heat map of differentially expressed genes across the whole transcriptome of *E. coli*. Columns represent genes and rows treatments with antibiotics at a concentration known to affect the cells. Z-score transformation was performed on mean log2 values (n = 3 replicates) for each gene, with blue denoting lower and red higher expression levels compared to the average. Hierarchical clustering of genes and samples is based on complete linkage and Pearson correlation distance. Color coded labels indicate compounds with similar MoA. (**b**) Correlation plot of mRNA expression levels from RNAseq with GFP intensity under the same gene promoter in *E. coli* strains quantified with HCS as the cell population median of the median pixel intensity per cell. (**c**) *E. coli* bacteria strains expressing recN and entC promoter GFP reporter gene constructs. Cells were treated with compound for 1h, and the individual images for membrane stain and GFP acquired with Opera QEHS instrument, 60x magnification. (**d**) Antibiotics treatment dose dependent intensity change in the GFP channel defined as fold standard deviation of non-treated samples for the median pixel intensity per cell, cell population median, N = 2, n = 2. (e) Heat map of differentially expressed reporter genes in *E. coli*. Columns represent genes and rows treatments with antibiotics at sublethal concentrations. Z-score transformation was performed on mean log2 values (N = 2, n = 1-2 replicates) for each gene, with blue denoting lower and red denoting higher expression levels compared to the average expression level. Hierarchical clustering of genes and samples is based on complete linkage and Pearson correlation distance. Color coded labels indicate compounds with similar MoA.

The absence of a clear morphological phenotypic fingerprint in the case of Nitroxoline prompted us to investigate the complementation of the morphological phenotype using features derived from molecular information. Therefore whole transcriptome sequencing (RNAseq) was performed on *E. coli* treated with a reference set of antibiotics at sublethal concentrations. Most of the tested compounds induced differential changes to the gene expression profiles in a reproducible manner. As in the case of the morphological phenotypic fingerprint, compounds with similar MoA tended to induce similar changes in gene expression that can be considered as molecular fingerprints (Fig. 5a).

Interestingly, compounds damaging DNA or acting on the ribosome led to drastic transcriptome perturbations, whereas compounds acting on cell wall synthesis had much less effect on the transcriptome.

Based on the expression changes observed in the RNAseq dataset, a panel of reporter genes was selected with the potential to distinguish the different reference MoAs. We used an *E. coli* library of fluorescent reporter strains in which green fluorescent protein (GFP) is transcriptionally fused to *E. coli* promoters for individual genes in order to study gene expression in a more high throughput manner, as RNAseq is relatively resource intensive (Fig. 5b). A linear correlation of GFP reporter intensity to RNAseq signal was observed for genes with basic expression down to around 100 Reads Per Kilobase per Million mapped reads (RPKM) as this threshold corresponds to the non-GFP related fluorescence signal. Approximately half of the initially selected reporter strains had a baseline expression level above the background signal, supporting that GFP strains could be used to report changes in gene expression upon antibiotic treatment. For example Nitroxoline but not Levofloxacin induced an increase in GFP intensity in a dose dependent way in the *entC* reporter strain and vice versa for the *recN* strain (Fig. 5c,d). Based on the expression change observed in the RNAseq dataset, a panel of GFP-reporter strains was selected, with the potential to still distinguish the different reference MoAs using a reduced gene set. The increased capacity enabled by a reduced gene set allowed a test of all compound in a dose response format, and the change in GFP intensity was extracted at the lowest concentration where more than 50% reduction in cell growth was observed across all strains and experiments. For each main class of antibiotics tested except Ceftriaxone, sets of signature genes were identified which allow clear identification of active compounds in a MoA specific manner as demonstrated using hierarchical clustering analysis (Fig. 5e).

### Subtle phenotypes can be recognized by ML-powered bacterial phenotypic characterization enabling the MoA analysis in bacterial species beyond *E. coli*

**Figure 6:**
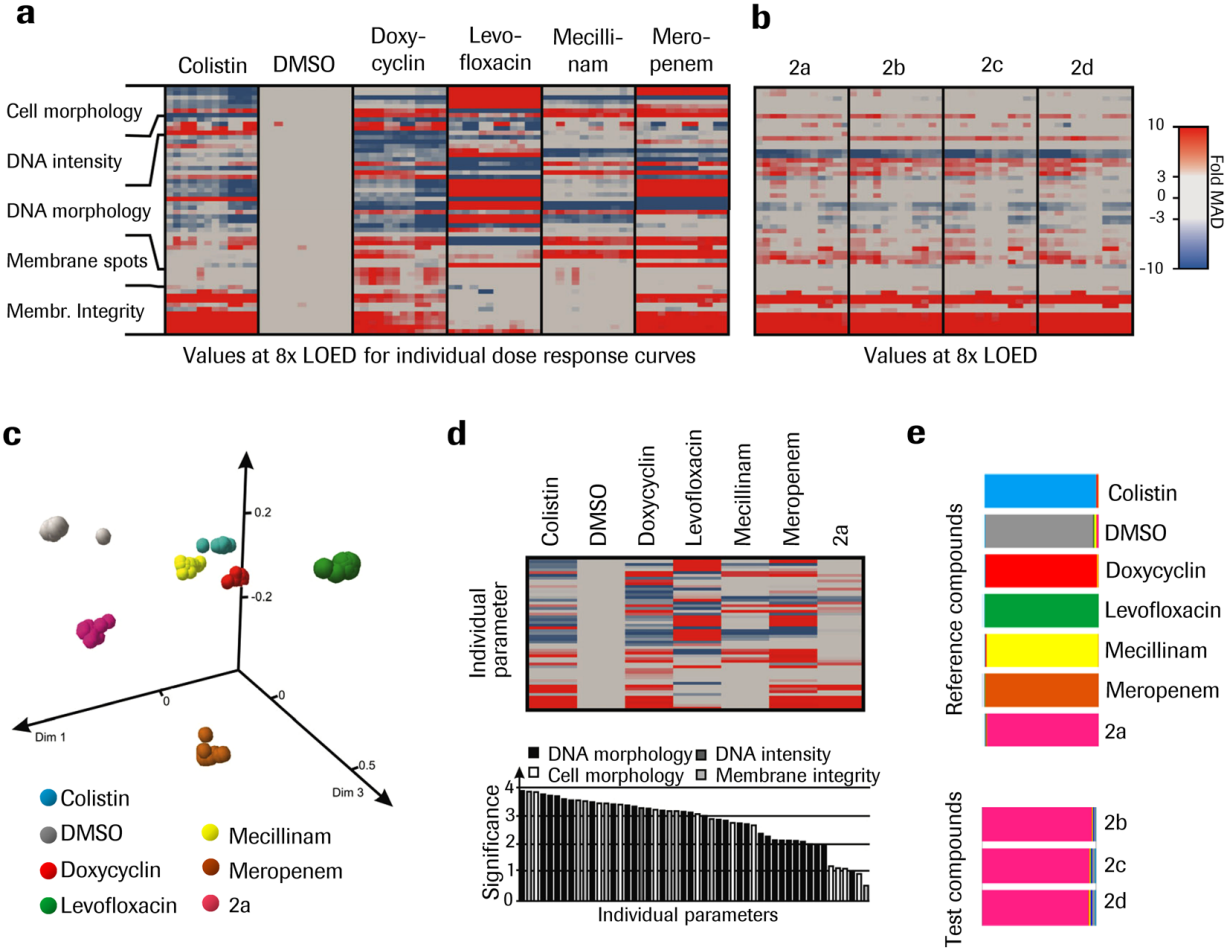
Adaptation to second species and application to novel antibiotics development (**a,b**) Compiled compound induced morphological changes at 8x LOED. For each compound, results from individual dose response curves from three experiments with LOED derived from the combined replicates within the experiment (n = 4) are shown. Each column corresponds to the data from one well and the rows individual features. The color indicates the change in the feature value, expressed as fold MAD over median calculated for all non-treated samples. The data are from a set of reference compounds (**a**), and a series of compounds with same chemical core-structure, (Tanimoto coefficients^29^ from 0.51 to 0.73) from an antibiotics development project with unknown MoA (**b**). (**c**) Similarity-based projection of the correlating random forest distance matrix into 3 dimensions showing clear separation between data points belonging to different compounds and close proximity of those belonging to the same compounds, forming clusters. For the Random forest analysis data from 4x and 8x LOED are pooled, and the number of datapoints are reduced by downsampling to be equal for all reference conditions. (**d**) Archetypes for the set of reference antibiotics (upper panel) with value calculation and heatmap scaling the same as in panel b, and distribution of feature importance contributing to archetypes (lower panel). (**e**) Out-of-bag validation of the random forest classification model for reference compound set (upper panel) and similarity score for different analogs from a new antibiotics series (lower panel).

To determine the potential to use the platform beyond *E. coli*, the common model species, we applied the protocol to a second species. *A. baumannii* cultures were exposed to a panel of reference antibiotics following the protocol optimized to *E. coli*. Interestingly, the detection of phenotypic changes to *A. baumannii* morphology was significantly more challenging for several reasons: wild-type *A. baumannii* cells are smaller, membrane integrity is weaker, morphologically more heterogeneous across the population, and the observable treatment induced phenotypic changes are less distinct. However with minor modifications to the protocol, it was possible to derive antibiotic specific fingerprints even for antibacterial compounds that display no apparent visible changes (Fig. 6a). This allowed to identify a new compound series originating from the primary library screen (series 2: **2a**–**2d**) This series, with potent activity against *A. baumannii*, which displayed a unique, well differentiated phenotypic fingerprint and was consistent across multiple analogs (Fig. 6b). Despite the reduced number of differential phenotypic features, it was possible to establish a random forest model that allowed a clear separation of the new series from the reference set as shown by the similarity-based projection into 3 dimensions (Fig. 6c). The series 2 displayed a unique archetype (Fig. 6d, upper panel). The relatively flat distribution of differential features shows that multiple parameters, as in the case of *E. coli*, are necessary to separate data points belonging to different compounds (Fig. 6d, lower panel). Remarkably, the *A. baumannii* optimized protocol enables an even better separation of the reference compounds in the 3D similarity-projection plots compared to *E. coli* (Fig. 6d, upper panel). All the series 2 test compounds displayed a high similarity score towards compound **2a** (Fig. 6e, lower panel) hence supporting the hypothesis that all 5 compounds might have the same molecular target and MoA.

## Discussion

In the present study we showed that a combined HCS and molecular information-based semi-automated phenotypic profiling platform, coupled to a ML-powered analysis pipeline, can effectively differentiate the MoA of novel antibacterial compound hits. Hence the known antibacterial compound and target space of an existing pharmaceutical compound library is expanded. In addition, our approach enables SAR guidance and increases confidence that chemical modifications introduced during SAR, in an attempt to improve potency, retain desired compound MoA.

Anti-infectives (antibiotics^9^, antivirals^10^ and anti-parasitic^11^) are traditionally a successful area for PDD due to the tight relation between disease model and readout. Killing bacteria in the multi-well plate corresponds quite closely to the sought pharmacodynamic (PD) and therapeutic effect in patients. However, this strength is conversely also the main limiting factor for the discovery of novel antibiotics. As our screening with a classical antibacterial susceptibility assay demonstrates, only a small fraction of the validated hits are sufficiently potent to allow target investigation in a traditional manner (spontaneous mutagenesis analysis at MIC and resistance generation through overexpression of potential targets). These results can be partially explained, as also observed by others^12,13^, by the limited chemical diversity present in pharmaceutical compound collections which are primarily established to penetrate and modulate biological processes in eukaryotic cells. In addition, in prokaryotic cells, small molecules are susceptible to active efflux by membrane-associated pumps^12,13^ further reducing bioactivity. Net permeability, the product of entry and efflux, is therefore a critical determinant of intrinsic sensitivity of bacterial species to antibacterials and dramatically limits the ability of a hit compound, especially those characterized by a suboptimal potency, to be identified in a classic antibacterial screen.

Recognizing this issue, we set to explore grey chemical matter, investigating potential for a possible source of new antibacterial drug development either with new resistance resistant scaffolds towards known targets or yet unknown targets. In doing that, we adopted a PDD approach inspired, amongst others, by the pioneering work by Nonejuie *et al*.^7^.

We first set to explore the phenotypic modulation induced by reference antibiotics commonly used in the clinics using HCS and transcriptomic via RNA-seq. The initial classification results demonstrated that additional data processing elements were needed to transform this basic phenotypic approach into an effective drug discovery tool. This resulted in the development of a data analysis pipeline composed of 3 consecutive parts: (a) the identification of the lowest concentration (LOED) for a test compound at which significant phenotypic changes are observed; (b) the development of a highly interactive user interface which allows full control over dataset curation, insuring consistency of the complex dataset and (c) the use of ML-based tools which allow generation of a similarity score for test compounds relative to a set of reference conditions.

We quickly recognized that rigorous identification of LOED was critical to correctly classify similarity between compounds with the same MoA but different level of antibacterial activity (*i.e*., with different MIC). To identify LOED reliably, we developed a spline fit-based approach, a powerful alternative to the traditional sigmoidal fit-based approaches normally used to determine dose dependency which are not particularly suited for the complex dose response effects observed in PDD.

The LOED captured the ability of a compound to induce a change in the individual feature and clustered the contributions in a consensus measurement. LOED proved to be a highly flexible tool capable of accommodating feature sets of different nature (*i.e*. derived by HCS or genomic analysis). Use of LOED enabled us to identify unique phenotypic fingerprints in *E. coli* for more than 10 different reference compounds, correlating with their corresponding MoA. Noteworthy, even subtle, but statistically robust, changes in some features, as in the case of kurtosis in the DNA intensity distribution, significantly contributed to the compound fingerprints. The adoption of ML data pipeline analysis methods allowed us to leverage all subtle changes and handle a complex multiparametric dataset in a robust manner, irrespective of the intrinsic variability observed in the individual measured parameters.

Application of the ML data pipeline to features, generated either by HCS or transcriptomic analysis, recognizes archetypical phenotypes toward which test compounds could be matched. Using that approach, we showed that the boronate series has the very same fingerprint as the FabI inhibitor Triclosan, a result supported by previous literature^14^. Our results show that fingerprint matching allows recognition of common structural elements present in multiple compounds, as it was the case for the 2-sulfonylated diazaborinine structural motif.

More importantly, the mismatch with archetypical phenotypes, as in the case of the series 2 active in *A. baumannii*, suggests that our approach allows identification of compounds with unknown MoA. Once recognized, those newly identified fingerprints can become part of the archetypic phenotype collection and be used to match close analogues generated by chemists. The approach therefore provides an exquisite and unique tool to track SAR, ascertaining whether molecular changes, introduced to enhance potency, result in a change of MoA and likely of target protein. This is a major improvement compared to classic PDD where MoA changes cannot be tracked.

We also realized that not all antibacterial effects can be captured by changes in morphology and explored the opportunity to expand the feature set with molecular information-derived genomic signature data. We investigated the molecular MoA of reference antibiotics using sequencing procedures. As in the case of HCS, molecular phenotype allowed correct clustering of compounds according to MoA and proved to be complementary to morphology-derived readouts, as shown in the case of Nitroxoline, which had a very clear molecular phenotype in absence of a morphological fingerprint. Vice versa some of the classical cell wall synthesis inhibitors, which induced strong morphological changes, displayed very limited molecular phenotypes. By including molecular features, the number of the distinguishable archetypic phenotypes can be further extended to cover additional MoAs. As we also show, fingerprints recognized by sequencing techniques could be reduced to a panel of reporter strains whilst retaining the capacity to correctly cluster compounds with a significant increase in throughput.

In addition to supporting hit MoA characterization and guiding SAR optimization, multiparametric high content screening (HCS) and genomic defined phenotypes have the potential to be applied in the context of a primary screen. This will enable a screen focused on identifying compounds with preferred MoA. Furthermore, phenotypic changes tend to appear at concentrations below the MIC, hence screening including these phenotypic readouts also has the potential to identify weak hits, incapable of inducing bacteria cell death or inhibiting bacteria growth at the screening concentration.

When evaluated in comparison to antibacterial susceptibility, LOED-delimited chemical space was shown to be larger than the MIC-defined space and partially correlated to it. The premise of our attempt is that those newly identified LOED active scaffolds would also qualify as valuable starting points for medicinal chemistry to be further transformed into MIC-active ones. This is particularly important when screening for phenotypic changes, as in the case of the series 2, identifying hits acting through a novel MoA amenable to medicinal chemistry.

In conclusion, we hope the present pilot work will facilitate and encourage, particularly the academic community, to scrutinize their unique compound collections using the presented alternative analysis paradigm aiming at identifying novel MoA-based *first in class* antibiotics from what today we disregard as non-attractive hits with limited or no MIC activity.

The approach and workflow presented here has been created with open source advanced analytic software and can be implemented and applied to any compound collection and likely to most bacteria species as our experiments with *E. coli* and *A. baumannii* demonstrated.

More generally, beyond the scope of the present study, we are convinced that such a combined multiparametric HCS/genomic approach coupled to ML could be profitably applied in PDD across a broad range of biological problems. Our proposed approach enables investigators to track the consistency of the MoA while improving the hit performance into a lead compound, something possible so far only in target based drug discovery. Beyond antibiotics, our approach might well be applied to rare diseases, where a complex phenotype is frequently the only certain disease relevant knowledge available to researchers as well as in complex diseases, such as RA or diabetes, where pointing to a single causal mechanism accounting for disease onset and progression is extremely difficult and where a combination of stimuli could at least partially mimic the disease phenotype.

## Materials and Methods

### Materials

#### Bacteria strains

*E. coli,* BW25113, CGSC Yale, Coli Genetic Stock Center, *A. baumannii*, ATCC 17978, Weizman library of GFP reporter strains: Dharmacon, GE healthcare and *E. coli ΔtolC* of the Keio single-gene deletion library^15^: Dharmacon, GE healthcare.

#### Assay plate

Corning 384 well black plate non-binding surface #3766. Antibiotics: Clarithromycin; Polymyxin B (AK Scientific Inc.; Union City; CA 94587; USA); Chloramphenicol; Doxycycline; Norfloxacin (Shore RoadHeysham; Lancashire UK); Avibactam, Ceftriaxone; Ciprofloxacin; Colistin; Levofloxacin; Mecillinam; Nisin; Nitrofurantoin; Nitroxoline; Triclosan, Trimethoprim (Sigma Aldrich; St. Gallen; Schweiz); 1a to 1o (custom synthesis, WuXi AppTec Shanghai China) AZ-LolCDE^16^; MD3^17^; GyrB/Par-ATPase^18^; CCCP (Cezomycin Carbonyl cyanide m-chlorophenyl hydrazone), 2a to 2d: In house synthesis, F. Hoffmann - La Roche, Basel, Switzerland; iso-sensitest medium; PBS without calcium and magnesium (Gibco by Lifetechnologies Europe, Zug, Switzerland), FM 4–64FX; SYTOX Green (Thermo Scientific by lifetechnologies Europe, Zug Switzerland, Dapi (Roche Diagnostics GMBH, Mannheim, Germany).

### Compound library screen and hit identification

The Roche pharma compound library contains over 4 million compounds. Prior to the antibacterial phenotypic screen, this number was reduced to 1.5 million by excluding compounds from series that were overrepresented and by balancing out the chemical diversity. The antibacterial activity e.g. inhibition of growth (OD600) of all compounds was tested in duplicates using a single concentration (40 µM) against the 4 Gram-Negative ESKAPE pathogens, i.e. *Acinetobacter baumannii* ATCC 17978, *Escherichia coli* BW25113, *Klebsiella pneumoniae* NCTC 13438, and *Pseudomonas aeruginosa* NCTC 11451. Around 10’000 compounds were identified that inhibit growth over 50% compared to untreated controls. The activity of these primary hits was further validated in a dose response screen (11 dilutions, 100 to 0.1 µM)(Fig. 1).

### Bacteria treatment with compound and preparation of samples for imaging

Two fold dilution series of compound was prepared in DMSO in a microtiter plate and diluted with isosensitest medium to 6% DMSO. 10 µl was transferred into each well in the assay plate. *E. coli* or *A. baumannii* bacteria were grown at 37 °C in iso-sensitest medium til ~OD 0.4 in exponential growthphase. The bacteria suspension was diluted to OD 0.2 with prewarmed medium, 20 µl pipetted into each well and incubated with compound for 1 h or 1 h 30 min (*E. coli* and *A. baumannii* respectively). 15 min before end of the incubation, 10 µl of 37 °C medium containing Sytox Green and FM-64 fluorescent dyes were added to a final concentration of 1 µM and 2.5 µg/ml respectively. At the end of the incubation period 40 µl PBS (room temperature) was added per well, the plate centrifuged for 1 min at 490 g and supernatant removed by aspiration to ~10 µl volume remaining per well. 10 µl 4% PFA in PBS containing 4 µg/ml DAPI was added and the samples incubated for 30 min at RT. The samples were washed by adding 60 ul PBS, centrifuging down and aspirating supernatant to 10 µl remaining volume. 50 µl PBS were added and 2.7 µl of the sample diluted into the imaging plate in PBS to final 30 µl volume. For *A. baumannii*, PBS was supplied with 4 µg/ml DAPI.

### Quantification of phenotypic changes with high content imaging and image analysis

Images of the samples for the three fluorescent stains where acquired using the Opera QEHS reader (Perkin-Elmer, Hamburg, Germany) equipped with a Nipkow Spinning disc for confocality using a 60x objective. Hoechst stain was imaged with laser excitation at 405 nm laser and a 455/50 nm band path (BP) filter for emission. The lipid membrane stain FM-64 was imaged using laser excitation at 488 nm, and a 565/40 nm/BP filter for emission and the Sytox stain with excitation at 488 nm and a 690/70 nm BP filter for excitation. 15 fields of view were imaged for each well, corresponding to ~0.23 mm^2^.

For phenotype characterization and quantification, an image analysis algorithm was developed for the instrument integrated software Acapella, generating a per-well derived cell population statistics comprising more than 100 features based on identification of cells and subcellular structures. Segmentation of individual cells was performed on the binary thresholded and converted membrane stain image, followed by noise removal based on area and multiple steps of morphological filling and a smoothing procedure with dilation and erosion. Cell clusters were identified and removed from subsequent analysis in multiple filtering steps: Touching objects upon dilation, cells exceeding a maximum area threshold or a length dependent width threshold and cells containing nucleoid clusters exceeding a width and area maximum. The membrane region was defined as an equidistant shift relative to the cell border. DNA cluster structures were identified independently of the cell outlines using the nuclei segmentation algorithm implemented in the software and then mapped back to the cell structures. More fine-granular segmentation of DNA clusters was obtained with the micronuclei module (DetectMicronucleiSP3) making use of a sliding parabola filter for background correction as well as texture filters for background and contrast filtering. Spot-like structures of the membrane were identified using the local intensity maxima followed by contrast and intensity selection steps (Spot_detection_C algorithm).

A default parameter setting for all segmentation modules were established using visual inspection to allow correct segmentation of a large range of phenotypes obtained upon treatment with reference compounds. Analysis of the experimental data was carried out on the Columbus database (Columbus™ Image Data Storage and Analysis system, PerkinElmer, Hamburg, Germany

A complete list of the features used for further analysis can be found in the Supplementary Materials and Method section, Table S1.

### Data processing

Statistical calculations and Machine Learning are carried out using R, both for LOED detection (batch mode) and from within Spotfire (Tibco Software Inc. Palo Alto, USA) by the user, i.e. interactively. Spotfire is attached to a relational database (Oracle Corporation, Redwood Shores, USA) which allows the user to dynamically add new data and annotations on the well level.

Quality control and curation of the data is carried out through custom spotfire templates with interactive annotation based on multiple visualizations of the data including direct link to original images. This allows the identification of wells with low cell number or other technical issues occurring during sample preparation, imaging or image analysis and systematic exclusion of poor quality data points from further analysis steps.

The LOED is identified for each combination of compound, concentration dilution series, bacterial strain and incubation time with n = 2–3 per experiment. The dataset consists of multiple readouts (features) normalized according to the MAD of the negative control samples on same plate. For each feature a dose-response relationship curve is fitted to the data, employing smoothing splines with maximum smoothness^19^. The process is illustrated in Supplemental Fig. 1. An LOED for the individual parameter (“individual LOED”) is determined if the spline’s 95% confidence interval completely exceeds the critical threshold of ±3x standard deviation of the negative control wells, relative to baseline of lowest concentrations (arrow-marked positions) and is defined as the concentration where this takes place. In case of no significant effect or technical failure for splinefit due to high noise level of the dataset no valid LOED will be derived for the individual feature. With individual LOEDs identified for at least three features, a consensus LOED is calculated using a weighted average of the individual LOEDs across all features with each feature’s contribution weighted by the goodness of its fit. To measure goodness of fit we use R^2^, the coefficient of determination, bounded to be between 0 and 1.

To reduce the shift of the LOED calculation towards higher concentrations caused by parameters where changes become significant at concentrations above the LOED, the data points from concentrations above 4x LOED are removed from the LOED identification in an iterative process. The statistic across experiments for the LOED is derived from the experiments for the individual compound, where the LOED is clearly within the dynamic range of the tested concentration range.

The quality of the fingerprint and consistency are assessed across the experiments used for a classification: Fingerprint quality: “Clear”: At least one parameter with >10 fold change in >85% of the individual data points at 4fold LOED. “Weak”, no single parameter with consistent change required for classification as “clear”, but visual inspection reveals presence of a systematic weaker fingerprint including >5 parameters. A heatmap visualization for the fold MAD distance to the DMSO well value for all parameters is used to verify that the fingerprint profile at the LOED is correctly identified and within the dynamic range of the dilution curve.

### Predictive modelling

For each compound a set of data points corresponding to the extracted values for the individual wells slightly above LOED are used for training the predictive model. During model training, the random forest algorithm^20^ is trained on a user-defined set of reference compounds, inducing clear and clearly distinct fingerprints, preferably covering different known modes of action. For each reference compound, the random forest algorithm infers a set of rules that associate patterns in the image readouts belonging to this compound with the associated compound label.

Model training involves finding suitable parameters for the “ntree” and “mtry” parameters, indicating the number of trees to grow in the forest, and the number of variables randomly sampled as candidates at each split of a decision tree in the forest, respectively. We keep ntree = 1000 constant and vary only mtry along three different values using the caret package^21^, with accuracy as metric function. For each mtry value, the model performance is estimated using out-of-bag (OOB) validation. The results are communicated to the user. Out-of-bag validation was shown to be an unbiased estimator of the mean accuracy^20^, as opposed to crossvalidation.

For training data, the compound label equals the compound name, i.e. we follow a multinomial classification setting. A trained random forest model returns the mutual similarity of any two training data points based on the RF proximity matrix, which is then used for identifying the compound-specific cluster centers of the training data (“archetypes”) and projecting the compound data points to 3-D using multidimensional scaling^22^. In addition, the overall variable importance, based on the Information Gain criterion calculated during rule inference, is derived.

After training, the resulting model can be queried to infer the similarity of the individual test compound data points to the set of reference compounds providing probabilities of similarities. A prediction returns a similarity distribution over the compound names used for training, indicating the similarity to any training compound. The similarity score for the individual test compounds is defined as the mean of the values for the individual data points.

### Data analysis for determining compound fingerprint similarity

For each random forest similarity score analysis, data points are combined from 2–3 independent experiments with the same compound set, each experiment with n = 2–3 to establish a random forest model. For *E. coli*, data points at 2x and 4x LOED are used, for *A. baumannii* 4x and 8x. The selected feature subset for each species is described in Supplementary Table S1.

As the first step in the classification of new test compounds on *E. coli* without known MoA, a similarity score was derived towards a default set of reference conditions: Colistin, Doxycycline, Ceftriaxone, Globomycin, Levofloxacin, Mecillinam and non-treated samples in the same experiment series. The quality of the model to predict difference and similarity was verified with the out of bag analysis, where a high score for the individual compound in classification of the samples used to build the model towards itself indicates a robust model. In our experience, a model where 0.7 or higher consistently is reached for all reference compounds towards them self is reliable for correct classification of test compounds. The exact value depends on strain, individual compound, parameter set and concentration shift relative to LOED.

The minimum fraction in the similarity score for a test compound MoA to likely be similar to one of the reference compounds was determined by analyzing a set of test compounds with the same and different MoA as the reference set.

The second step in the analysis is a series of similarity classifications custom to the set of test and additional reference compounds in the experiment. In a consecutive manner, the compounds are included one at a time in the set of reference compounds used to build the model by replacing one of the reference compounds. The reference compound is selected based on the fingerprint similarity to the test compound in such a way that all compounds in the reference set still have clearly distinct fingerprints. For a test compound to finally be classified as having a high similarity towards another compound, it should have a high similarity score in the resulting matrix analysis both when run as test and as a part of the reference set.

Preferably a small set of test compound analogs are classified in parallel, where a mutual high similarity score in the matrix, support that the fingerprint is the result of a single MoA.

### RNA-sequencing

*E. coli* bacteria were cultured in isosensitest-medium in the absence or presence of different antibiotics for ~ 25 min till ~OD 0.2 at concentrations expected to be sublethal (AZ-LolCDE: 200 µM; a1: 50 µM; Ceftriaxone: 0.75 µM; Chloramphenicol: 25 µM; Ciprofloxacin: 1 µM; Clarithromycin: 50 µM; Colistin: 0.13 µM; Doxycycline: 7.5 µM; Globomycin: 25 µM; Levofloxacin: 0.75 µM; Mecillinam: 5 µM; Meropenem: 0.78 µM; Nitrofurantoin: 50 µM; Nitroxoline: 100 µM; Norfloxacine: 1 µM; Polymyxin B: 0.25 µM; Trimethoprin: 50 µM).

After the treatment bacteria from 2 ml culture were isolated with centrifugation and resuspended in Qiagen RNAprotect Bacteria Reagent (Qiagen Inc., USA), incubated for 5 min, centrifuged, and flash frozen on dry ice. Total RNA was extracted by incubating bacteria in Enzymatic Lysis Buffer (lysozyme & proteinase K) for 5 min followed by addition of Qiagen RLT Lysis Buffer and RNA purification using the Qiagen RNeasy Mini kit combined with DNase treatment on a solid support (Qiagen Inc., USA). Template DNA molecules suitable for sequencing were prepared from 1000 ng of total RNA. First, bacterial ribosomal RNA was depleted using the Ribo-Zero Magnetic Kit Bacteria (Illumina Inc., San Diego, CA). After depletion, RNA was resuspended in TruSeq Total RNA Sample Prep Kit Fragmentation buffer (8.5 µl RNA and 8.5 µl buffer) and reverse transcribed into cDNA using random hexamer primer. Then cDNA was further processed for the construction of sequencing libraries according to the manufacturer’s recommendations using the TruSeq Stranded mRNA Sample Prep Kit (Illumina Inc., San Diego, CA). After 14 cycles of PCR amplification, the size distribution of the barcoded DNA libraries was estimated by electrophoresis on Agilent High Sensitivity Bioanalyzer microfluidic chips. Minimum sizes of amplified libraries were determined as >200 nucleotides and average sizes of ~340 nucleotides. Libraries were quantified using the KAPA Library Quantification Kit (Kapa Biosystems, Boston, MA). Barcoded Libraries were randomized and pooled at equimolar concentrations, spiked with 1% PhiX control library, and diluted to 20 pM prior to loading onto the flow cell of an Illumina HiSeq2500 instrument (Illumina Inc., San Diego, CA) for both clustering and sequencing. Libraries were extended and bridge amplified to create single sequence clusters using the HiSeq PE Cluster Kit v4 cBot HS (Illumina Inc., San Diego, CA). The flow cell carrying amplified clusters was then sequenced in high output run mode with 50 cycles for read 1, 7 cycles for the barcode index and 50 cycles for read 2 using the HiSeq SBS Kit v4 chemistry (Illumina Inc., San Diego, CA). 2 × 50-bp paired-end reads were generated with ~11 million read pairs per sample. Real time image analysis and base calling was performed on the HiSeq2500 with the HiSeq Control Software v2.2.37. CASAVA software version 1.8.2 was used for demultiplexing and production of FASTQ sequence files.

The RNA-sequencing data from this study has been deposited at the NCBI Gene Expression Omnibus (http://www.ncbi.nlm.nih.gov/geo) under the accession number GSE110137.

### Bioinformatic analysis of RNA-sequencing data

To estimate gene expression levels, paired-end RNA-seq reads were mapped onto the *E. coli* genome (GenBank: U00096) by using the short read aligner GSNAP and Genbank/EcoGene transcript annotations^23,24^. Mapped reads for all gene transcripts (counts) were combined into a single value, normalized and denoted as RPKMs (number of mapped reads per kilobase transcript per million mapped reads)^25^. Heat map visualization was performed in R.

A subset of significantly changed genes was selected based on the fold change in RPKM with the potential to still detect compound specific changes in expression level and discriminate as many of the MoAs in the compound set as possible. Corresponding *E. coli* reporter cell lines, expressing GFP under the individual gene promoter were selected from the Weizman-library^26,27^. Following the compound induced changes to GFP intensity for the individual lines was determined in a dose dependent manner using the same image based approach as for morphological characterization, with the key readout being the cell population median of intensity in the GFP channel normalized to individual cell area. Incubation with compound was extended to 1.5 h. Since not all compounds induce morphological changes, the concentration used for comparing molecular profile of the individual compounds was selected based on reduction in cell number. for the POC classification, the lowest individual compound concentration where the median cell number across all experiments were reduced more than 50% relative to negative control (AZ-LolCDE: 200 µM; a1: 25 µM; Ceftriaxone: 0.047 µM; Chloramphenicol: 6.3 µM; Ciprofloxacin: 0.39 µM; Clarithromycin: 25 µM; Colistin: 1 µM; Doxycycline: 3.8 µM; Globomycin: 0.78 µM; Levofloxacin: 0.094 µM; Mecillinam: 2.5 µM (no concentrations with cell number reduction below 50% up to 10 µM, use concentration with highest frequency of reporterstrains with GFP intensity change); Meropenem: 3.1 µM; Nitrofurantoin: 25 µM; Nitroxoline: 50 µM; Norfloxacine: 0.39 µM; Polymyxin B: 1 µM; Trimethoprin: 0.78 µM). Hierarchical clustering of both, rows (compounds) and reporter genes (columns), was performed by using the Euclidean distance and the Ward’s method^28^.

## Supplementary information

Supplementary information

## Data Availability

The RNA-sequencing data from this study has been deposited at the NCBI Gene Expression Omnibus (http://www.ncbi.nlm.nih.gov/geo) under the accession number GSE110137. Data used to generate the similarity score are available upon reasonable request from the corresponding author. A description of the quantified parameters is available in the supplementary section to allow users of any suitable imaging device and image analysis software to generate comparable dataset suitable for classification.
