# Supplementary material for: Machine learning-powered antibiotics phenotypic drug discovery
Source: Sci Rep. 2019 Mar 21;9:5013. doi: 10.1038/s41598-019-39387-9 (PMC6428806; doi:10.1038/s41598-019-39387-9)
Supplement: Supplementary file 1 — Supplementary information [file 41598_2019_39387_MOESM1_ESM.pdf]

Supplementary information

Machine learning-powered antibiotics phenotypic drug discovery

Sannah Zoffmann[1,*], Maarten Vercruysse[1], Fethallah Benmansour[1], Andreas Maunz[1], Luise Wolf[1], Rita Blum Marti[1], Tobias Heckel[1], Haiyuan Ding[2], Hoa Truong[1,§], Michael Prummer[1,†], Roland Schmucki[1], Clive S. Mason[3], Kenneth Bradley[1], Asha Ivy Jacob[1], Christian Lerner[1], Andrea Araujo del Rosario[1], Mark Burcin[1], Kurt Amrein[1] and Marco Prunotto[1,‡]

Figure-S1

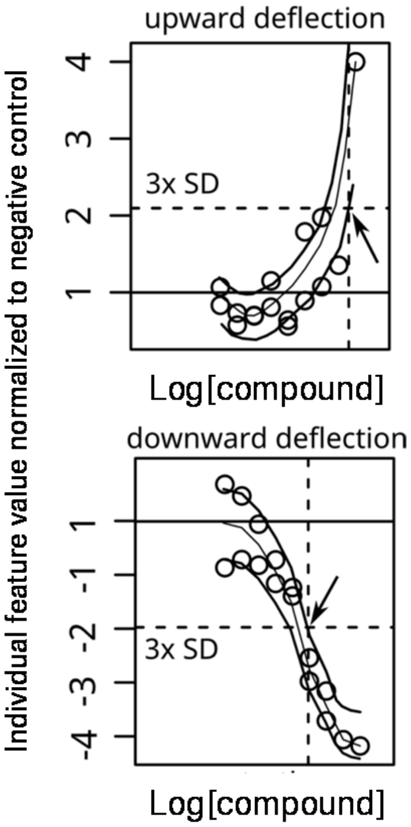

Figure-S1 - Deriving LOED

Example of a feature spline fit's 95% confidence interval completely exceeding (arrow) the critical thresholds of 1 +/- 3x standard deviation of DMSO (dashed horizontal). Top panel: Individual LOED (dashed vertical) detected by upward deflection. Lower panel: Individual LOED (dashed vertical) detected by downward deflection

Table-S1

Feature in E. coli classification, (value figure 2C)	Category	Segmented object(s)	Stain (for intensity)	feature	calculation	feature calculation	population analysis	selection for A. baumanii classification
1	DNA morphology	Whole cell/DNA sub-structure 2		count	objects per cell	2 object counts per cell	fraction	x
2 (3)	Cell morphology	whole cell		area	None	area	mean	x
3	Cell morphology	whole cell		area	None	area	median	x
4	Quality control	cell cluster/initially segmented cells		area	None	area fraction	mean per field	
5 (1)	Cell morphology	whole cell		eccentricity	None	eccentricity	median	x
6	DNA morphology	whole cell	DAPI	intensity (kurtosis)	None	intensity kurtosis	median	x
7	Membrane integrity	whole cell	Sytox	intensity(kurtosis)	None	intensity kurtosis	median	x
8	Cell morphology	whole cell		length	None	length	median	x
9 (8)	Membrane intensity	whole cell	membrane	intensity	mean	intensity mean	median	
10	Membrane intensity	whole cell	membrane	intensity	sum	intensity sum	median	
11	DNA intensity	whole cell	DAPI	intensity	mean	intensity mean	median	
12	DNA intensity	whole cell	DAPI	intensity	sum	intensity sum	median	
13	Cell morphology	whole cell		perimeter	None	perimeter	median	x
14	Quality control	whole cell		area/width	None	area/width	fraction	
15	Quality control	whole cell		clustering	None	clustering	fraction	
16 (2)	Cell morphology	whole cell		shapefactor/roundness	None	shapefactor/roundness	median	x
17	DNA morphology	whole cell	DAPI	intensity (skewness)	None	intensity skewness	median	
18	Membrane integrity	whole cell	Sytox	intensity (skewness)	None	intensity skewness	median	x
19	Membrane integrity	whole cell	Sytox	intensity	mean	intensity mean	median	x
20	Membrane integrity	whole cell	Sytox	intensity	sum	intensity sum	median	
21	Cell morphology	whole cell		width	None	width	median	x
22	Quality control	initially segmented cells		area	None	area	median	
23	Quality control	initially segmented cells		length	None	length	median	
24	Quality control	initially segmented cells		width	None	width	median	
25	DNA morphology	whole cell/DNA sub-structure 1		presence of DNA cluster	None	0 object count per cell	fraction	
26	DNA morphology	whole cell/DNA sub-structure 1		presence of DNA cluster	None	0 object count per cell	mean per field	
27	Membrane integrity	DNA sub-structure 1	Sytox	intensity above threshold	None	average intensity above threshold	fraction	
28	DNA morphology	whole cell/DNA sub-structure 1		area	None	ratio DNA cluster area/cell area	median	x
29	Membrane integrity	whole cell	Sytox	intensity above threshold	None	average intensity above threshold	fraction	
30	Membrane integrity	DNA sub-structure 1	Sytox	intensity above threshold	None	average intensity above threshold	fraction	
31	Membrane spots	whole cell/membrane/spot structure	membrane	Integrated background subtracted spot intensity in cell area per membrane area	mean	Integrated background subtracted spot intensity in cell area per membrane area	mean	
32	Membrane spots	whole cell/membrane/spot structure	membrane	subtracted spot intensity in cell area per integrated signal in membrane area	mean	Integrated background subtracted spot intensity in cell area per integrated signal in membrane area	mean	x
33	Membrane spots	whole cell/membrane/spot structure	membrane	Integrated spot intensity in cell area per integrated signal in membrane area	mean	Integrated spot intensity in cell area per integrated signal in membrane area	mean	x
34	Membrane spots	whole cell/membrane/spot structure	membrane	Integrated spot intensity in cell area per membrane area	mean	Integrated spot intensity in cell area per membrane area	mean	

Table-S1 continued

35	Quality control	membrane	membrane	intensity		pixel nr above threshold in membrane image defined as 0.8 x median of membrane image intensity normalized to total membrane region	none	
36	Membrane intensity	membrane	membrane	intensity	mean	intensity mean	median	
37	Membrane intensity	membrane	membrane	intensity	sum	intensity sum	median	
38	DNA intensity	membrane	DAPI	intensity	mean	intensity mean	median	
39	DNA intensity	membrane	DAPI	intensity	sum	intensity sum	median	
40	Membrane integrity	membrane	Sytox	intensity	mean	intensity mean	median	x
41	Membrane integrity	membrane	Sytox	intensity	sum	intensity sum	median	
42	DNA morphology	Whole Cell/DNA sub-structure 2		distance	mean	distance mean from cell masscentre per cell	median	
43	DNA morphology	Whole Cell/DNA sub-structure 2		eccentricity	mean	eccentricity mean per cell	median	x
44	DNA morphology	Whole Cell/DNA sub-structure 2		length	mean	length mean per cell	median	x
45	DNA morphology	Whole Cell/DNA sub-structure 2		count	sum	object count per cell	mean	x
46	DNA morphology	Whole Cell/DNA sub-structure 2		count	sum	object count per cell	median	
47	DNA morphology	Whole Cell/DNA sub-structure 2		perimeter	mean	perimeter mean per cell	median	
48	DNA morphology	Whole Cell/DNA sub-structure 2		shapefactor/roundness	mean	shapefactor/roundness mean per cell	median	x
49	DNA morphology	Whole Cell/DNA sub-structure 2		area	sum	area sum per cell	median	x
50	DNA morphology	Whole Cell/DNA sub-structure 2		width	mean	width mean per cell	median	x
51	DNA morphology	Whole Cell/DNA sub-structure 2		count	objects per cell	1 object count per cell	fraction	x
52	DNA morphology	DNA sub-structure 1		area	None	area	mean	x
53	DNA morphology	whole cell/DNA sub-structure 1		area	pixel sum	area pixel sum	median	x
54	DNA morphology	DNA sub-structure 1		area	None	area	median	x
55 (6)	DNA morphology	whole cell/DNA sub-structure 1		area	pixel sum	area pixel sum	median	x
56	Quality control	DNA sub-structure 1		area	None	area	mean	
57	DNA morphology	DNA sub-structure 1	DAPI	contrast	None	contrast	mean	
58	DNA morphology	whole cell/DNA sub-structure 1	DAPI	contrast	mean	contrast mean per cell	median	
59	DNA morphology	DNA sub-structure 1		density	None	density	median	x
60	DNA morphology	DNA sub-structure 1		eccentricity	None	eccentricity	median	x
61	DNA morphology	whole cell/DNA sub-structure 1		eccentricity	mean	eccentricity mean per cell	median	x
62	DNA intensity	DNA sub-structure 1	DAPI	intensity(kurtosis)	None	intensity kurtosis	median	x
63	Membrane integrity	DNA sub-structure 1	Sytox	intensity(kurtosis)	None	intensity kurtosis	median	x
64	DNA morphology	DNA sub-structure 1		length	None	length	median	x
65	DNA morphology	whole cell/DNA sub-structure 1		length	mean	length mean per cell	median	x
66	DNA morphology	whole cell/DNA sub-structure 1	DAPI	intensity(kurtosis)	mean	intensity kurtosis mean per cell	median	x
67	DNA intensity	whole cell/DNA sub-structure 1	DAPI	intensity(skewness)	mean	intensity skewness mean per cell	median	x
68 (4)	DNA intensity	whole cell/DNA sub-structure 1	DAPI	average intensity	mean	average intensity mean per cell	median	
69	DNA intensity	whole cell/DNA sub-structure 1	DAPI	total intensity	mean	total intensity mean per cell	median	
70	DNA intensity	whole cell/DNA sub-structure 1	DAPI	average intensity	sum	average intensity sum per cell	median	
71	Quality control	DNA sub-structure 1		count	None	count DNA sub-structures in DNA clusters (touching)	fraction	
72	Quality control	DNA sub-structure 1	DAPI	count	None	count isolated DNA sub-structures	fraction	
73	DNA morphology	whole cell/DNA sub-structure 1	DAPI	count	objects per cell	count objects per cell	mean	x
74	DNA morphology	whole cell/DNA sub-structure 1	DAPI	count	objects per cell	count objects per cell	median	

Table-S1 continued

75	Quality control	DNA sub-structure 1	DAPI	count	None	average count per DNA cluster	mean	
76	DNA morphology	DNA sub-structure 1	DAPI	perimeter	None	perimeter	median	
77	DNA morphology	whole cell/DNA sub-structure 1	DAPI	perimeter	mean	perimeter mean per cell	median	x
78	DNA intensity	DNA sub-structure 1	DAPI	average intensity	None	average intensity	median	
79	DNA intensity	DNA sub-structure 1	DAPI	total intensity	None	total intensity	median	
80	Membrane intensity	Equidistant Ring around DNA sub-structure 1	membrane	average intensity	None	average intensity	median	
81	Membrane intensity	Equidistant Ring around DNA sub-structure 1	membrane	total intensity	None	total intensity	median	
82	Membrane intensity	whole cell/Equidistant Ring around DNA sub-structure 1	membrane	average intensity	mean	average intensity mean per cell	median	
83	Membrane intensity	whole cell/Equidistant Ring around DNA sub-structure 1	membrane	total intensity	mean	total intensity mean per cell	median	
84	Membrane intensity	whole cell/Equidistant Ring around DNA sub-structure 1	membrane	average intensity	pixel sum	average intensity pixel sum per cell	median	
85	DNA intensity	Equidistant Ring around DNA sub-structure 1	DAPI	average intensity	None	average intensity	median	
86	DNA intensity	Equidistant Ring around DNA sub-structure 1	DAPI	total intensity	None	total intensity	median	
87	DNA intensity	whole cell/Equidistant Ring around DNA sub-structure 1	DAPI	average intensity	mean	average intensity mean per cell	median	
88	DNA intensity	whole cell/Equidistant Ring around DNA sub-structure 1	DAPI	total intensity	mean	total intensity mean per cell	median	
89	DNA intensity	whole cell/Equidistant Ring around DNA sub-structure 1	DAPI	average intensity	pixel sum	average intensity pixel sum per cell	median	
90	DNA morphology	DNA sub-structure 1		shapefactor/roundness	None	shapefactor/roundness	median	x
91	DNA morphology	whole cell/DNA sub-structure 1		shapefactor/roundness	mean	shapefactor/roundness mean per cell	median	x
92 (5)	DNA intensity	DNA sub-structure 1	DAPI	intensity(skewness)	None	intensity(skewness)	median	x
93	Membrane integrity	DNA sub-structure 1	Sytox	intensity(skewness)	None	intensity(skewness)	median	x
94	Membrane integrity	DNA sub-structure 1	Sytox	average intensity	None	average intensity	median	x
95	Membrane integrity	DNA sub-structure 1	Sytox	total intensity	None	total intensity	median	
96	Membrane integrity	whole cell/DNA sub-structure 1	Sytox	average intensity	mean	average intensity mean per cell	median	x
97	Membrane integrity	whole cell/DNA sub-structure 1	Sytox	total intensity	mean	total intensity mean per cell	median	
98	Membrane integrity	whole cell/DNA sub-structure 1	Sytox	average intensity	pixel sum	average intensity pixel sum per cell	median	
99	DNA morphology	whole cell/DNA sub-structure 1	Sytox	intensity(kurtosis)	mean	intensity(kurtosis) mean per cell	median	
100	Membrane integrity	whole cell/DNA sub-structure 1	Sytox	intensity(skewness)	mean	intensity(skewness) mean per cell	median	
101	DNA morphology	DNA sub-structure 1		width	None	width	median	x
102	DNA morphology	whole cell/DNA sub-structure 1		width	mean	width mean per cell	median	x
103	Quality control	whole cell/DNA sub-structure 1		count	None	count per cell	fraction	
104	Quality control	whole cell/DNA sub-		count	None	count per cell	mean per	
105	Membrane spots	whole				number of spots per	mean	x
106	Membrane spots	whole				number of spots per	mean	x
107	Membrane integrity	whole cell	Sytox	intensity above threshold	None	average intensity above threshold	fraction	
108	DNA morphology	Whole Cell/DNA sub-structure 2		count	objects per cell	>2 object counts per cell	fraction	x
109	DNA intensity	DNA sub-structure 1	DAPI	total intensity	pixel sum	total intensity in DNA sub-structure 1 relative to total intensity in cell	median	x
110 (7)	Membrane integrity	whole cell	Sytox	average intensity	None	average intensity	mean	x
111	Membrane integrity	DNA sub-structure 1	Sytox	average intensity	None	average intensity	mean	x
112	DNA morphology	Whole Cell/DNA sub-structure 2		count	objects per cell	0 object count per cell	fraction	x

Table-S1: *Individual image derived features used for Random Forest analysis*

Table-S1 describes the calculation of the individual feature values based on the underlying segmented objects, the stain used to extract the feature value, the type of feature calculation as well as the measure used for population analysis. The subset of features utilized for *A. baumanii* Random Forest classification are indicated by x. Segmented objects used for feature extraction comprised whole cells and subcellular structures on their own or mapped back to whole cells. Smaller DNA structures named DNA sub-structure 2 , identified with a dedicated Micronuclei algorithm were always mapped back to the whole cells resulting in features summarized within cells and across the entire cell population per well. Nucleoid features were further described by an equidistant region in DNA clusters 1 and furthermore spot structures in the membrane region. Besides morphological features, intensity-based features were based on the three stains, indicated in the stain column. Measures derived from the segmented object are described in the feature calculation. The summary statistics used for the population analysis outlines the per well calculated value based on the entirety of segmented objects.

Table-S2

E. *Coli*																					
		Reproducible		CEFTRIAXONE		COLISTIN		DOXYCYCLINE		GLOBOMYCIN		LEVOFLOXACIN		MECILLINAM		DMSO		Analysis	LOED		
Antibacterial	MOA	fingerprint		median	stdev	median	stdev	median	stdev	median	stdev	median	stdev	median	stdev	median	stdev	count	median	Stdev	count
Reference antibiotics																					
Ceftriaxone	Cell wall	clear	*out of bag analysis*	**0.89**	*0.06*	**0.02**	*0.01*	**0.01**	*0.01*	**0.01**	*0.01*	**0.04**	*0.02*	**0.01**	*0.00*	**0.01**	*0.01*	12	-7.6	*0.2*	25
Colistin	membrane integrity	clear		**0.01**	*0.01*	**0.90**	*0.04*	**0.03**	*0.01*	**0.02**	*0.01*	**0.02**	*0.01*	**0.01**	*0.01*	**0.01**	*0.01*		-6.3	*0.3*	25
Doxycycline	Ribosome	clear		**0.02**	*0.01*	**0.02**	*0.01*	**0.88**	*0.05*	**0.02**	*0.01*	**0.04**	*0.04*	**0.01**	*0.01*	**0.02**	*0.01*		-5.7	*0.6*	24
Globomycin	Lipoprotein export	clear		**0.01**	*0.01*	**0.02**	*0.03*	**0.01**	*0.01*	**0.89**	*0.05*	**0.01**	*0.01*	**0.04**	*0.02*	**0.00**	*0.00*		-5.2	*0.6*	24
levofloxacin	Topoisomerase	clear		**0.04**	*0.04*	**0.01**	*0.01*	**0.04**	*0.02*	**0.01**	*0.01*	**0.88**	*0.06*	**0.01**	*0.01*	**0.01**	*0.00*		-7.3	*0.7*	24
Mecillinam	Cell wall	clear		**0.00**	*0.00*	**0.01**	*0.01*	**0.01**	*0.01*	**0.03**	*0.01*	**0.01**	*0.01*	**0.90**	*0.04*	**0.02**	*0.02*		-6.2	*0.4*	24
DMSO	DMSO			**0.01**	*0.01*	**0.02**	*0.02*	**0.04**	*0.02*	**0.00**	*0.00*	**0.01**	*0.01*	**0.04**	*0.02*	**0.88**	*0.06*				
Test antibiotis: Reference MOA similar			Similarity score towards reference antibiotics																		
Cefepim	Cell wall	clear		**0.71**	*0.05*	**0.03**	*0.02*	**0.03**	*0.03*	**0.03**	*0.01*	**0.14**	*0.07*	**0.04**	*0.03*	**0.02**	*0.01*	5	-7.5	*0.8*	9
Polymyxin_B	membrane integrity	clear		**0.04**	0.02	**0.61**	0.25	**0.06**	0.04	**0.08**	0.01	**0.03**	0.03	**0.03**	0.06	**0.14**	0.16	4	-6.5	0.9	9
Clarithromycin	Ribosome	clear		**0.01**	*0.03*	**0.03**	*0.05*	**0.67**	*0.07*	**0.02**	*0.02*	**0.10**	*0.05*	**0.04**	*0.03*	**0.03**	*0.05*	3	-4.9	*0.3*	4
Chloramphenicol	Ribosome	clear		**0.01**	0.00	**0.04**	0.03	**0.76**	0.01	**0.03**	0.01	**0.04**	0.01	**0.06**	0.03	**0.06**	0.02	2	-4.9	0.2	5
AZ-LolCDE	Lipoprotein export	no 4xLoED		***NA***	*NA*	***NA***	*NA*	***NA***	*NA*	***NA***	*NA*	***NA***	*NA*	***NA***	*NA*	***NA***	*NA*	*NA*	-4.0	*0.1*	8
Ciprofloxacin	Topoisomerase	clear		**0.12**	*0.05*	**0.04**	*0.02*	**0.08**	*0.06*	**0.02**	*0.01*	**0.69**	*0.13*	**0.02**	*0.01*	**0.01**	*0.03*	5	-7.5	*0.2*	7
Norfloxacin	Topoisomerase	clear		**0.12**	*0.07*	**0.01**	*0.01*	**0.13**	*0.03*	**0.01**	*0.00*	**0.72**	*0.06*	**0.01**	*0.01*	**0.01**	*0.01*	4	-7.1	*0.2*	3
Test antibiotics: Unknown or different MOA																					
Avibactam	b-lactamase, Cell wall	clear		**0.02**	0.01	**0.04**	0.03	**0.05**	0.03	**0.09**	0.05	**0.01**	0.03	**0.61**	0.09	**0.18**	0.09	5	-5.2	0.4	9
CCCP	Membrane potential	biphasic		***NA***	*NA*	***NA***	*NA*	***NA***	*NA*	***NA***	*NA*	***NA***	*NA*	***NA***	*NA*	***NA***	*NA*	*NA*	NA		
GyrB/ParE ATPas‹	Topoisomerase	clear		**0.07**	0.06	**0.08**	0.01	**0.28**	0.10	**0.02**	0.00	**0.39**	0.09	**0.03**	0.01	**0.13**	0.12	2	-6.5	*0.1*	5
MD3	SPase1	no 4xLoED		***NA***	*NA*	***NA***	*NA*	***NA***	*NA*	***NA***	*NA*	***NA***	*NA*	***NA***	*NA*	***NA***	*NA*	*NA*	-4.0	*0.4*	9
Nitrofurantoin	unknown	clear		**0.11**	0.04	**0.10**	0.04	**0.24**	0.12	**0.08**	0.02	**0.39**	0.09	**0.06**	0.06	**0.04**	0.03	4	-5.2	*0.1*	8
Nitroxoline	unknown	no		***NA***	*NA*	***NA***	*NA*	***NA***	*NA*	***NA***	*NA*	***NA***	*NA*	***NA***	*NA*	***NA***	*NA*	*NA*	NA		
Triclosan	FabI	clear		**0.04**	0.04	**0.29**	0.17	**0.09**	0.16	**0.18**	0.08	**0.08**	0.05	**0.06**	0.12	**0.17**	0.07	3	-4.9	*0.6*	6
Trimethoprim	DNA synthesis	clear		**0.09**	0.02	**0.06**	0.02	**0.22**	0.10	**0.10**	0.02	**0.29**	0.12	**0.11**	0.02	**0.09**	0.10	4	-6.0	*0.5*	8

Table-S2 Similarity score for known antibacterial compounds on E coli WT

Similarity score derived with Random forest analysis from compiled phenotypic changes quantified across multiple experiments. The Random Forrest model is established using the parameter set for the six reference antibiotics and non-treated samples using the values at 2 and 4 fold LOED. Each analysis combines values from 2-3 experiments with n=2-3. The number of data points for each reference condition is reduced to a similar number by automated selection of data points for the individual experiments. The similarity score in the individual experiment is the frequency of matching prediction expressed as a fraction of 1, where a higher value represents a higher similarity.

Fingerprint quality: “Clear”: At least one parameter with >10 fold change in >85% of the individual datapoints at 4fold LOED. “Weak”, no single parameter with consistent change required for classification as “clear”, but visual inspection reveals presence of a systematic weaker fingerprint including >5 parameters. LOED is derived for each individual experiment with n=1-3.

Table-S3

E. coli ΔTolC		Standard E. coli panel of reference compounds							Additional compounds included in reference set by replacement									
Test compounds		CEFTRIAXONE	COLISTIN	DOXYCYCLINE	GLOBOMYCIN	LEVOFLOXACINE	MECILLINAM	DMSO	GyrB/ParE ATPase	MD3	NITROFURANTOIN	TRICLOSAN	TRIMETHOPRIM	1a	1b	1h	1j	1o
**CEFTRIAXONE**	median	**0.90**	**0.02**	**0.01**	**0.01**	**0.05**	**0.01**	**0.01**	**0.04**	**0.00**	**0.07**	**0.03**	**0.04**	**0.02**	**0.03**	**0.02**	**0.00**	**0.01**
	stdev	*0.04*	*0.01*	*0.01*	*0.01*	*0.01*	*0.00*	*0.01*	*0.03*	*0.00*	*0.02*	*0.01*	*0.02*	*0.03*	*0.03*	*0.02*	*0.00*	*0.00*
	N	*5*	*5*	*5*	*5*	*5*	*5*	*5*	*3*	*4*	*4*	*5*	*2*	*5*	*5*	*5*	*3*	*5*
**COLISTIN**	median	**0.02**	**0.90**	**0.01**	**0.03**	**0.01**	**0.01**	**0.02**	**0.02**	**0.01**	**0.04**	**0.04**	**0.03**	**0.04**	**0.04**	**0.04**	**0.01**	**0.01**
	stdev	*0.01*	*0.04*	*0.01*	*0.01*	*0.01*	*0.00*	*0.01*	*0.04*	*0.01*	*0.03*	*0.02*	*0.01*	*0.03*	*0.02*	*0.01*	*0.00*	*0.01*
	N	*5*	*5*	*5*	*5*	*5*	*5*	*5*	*3*	*4*	*4*	*5*	*2*	*5*	*5*	*5*	*3*	*5*
**DOXYCYCLINE**	median	**0.01**	**0.01**	**0.92**	**0.00**	**0.03**	**0.01**	**0.02**	**0.17**	**0.02**	**0.08**	**0.01**	**0.07**	**0.01**	**0.01**	**0.01**	**0.03**	**0.03**
	stdev	*0.01*	*0.00*	*0.02*	*0.00*	*0.01*	*0.00*	*0.01*	*0.08*	*0.02*	*0.04*	*0.01*	*0.02*	*0.01*	*0.01*	*0.01*	*0.01*	*0.02*
	N	*5*	*5*	*5*	*5*	*5*	*5*	*5*	*3*	*4*	*4*	*5*	*2*	*5*	*5*	*5*	*3*	*5*
**GLOBOMYCIN**	median	**0.01**	**0.01**	**0.01**	**0.95**	**0.01**	**0.02**	**0.00**	**0.01**	**0.01**	**0.02**	**0.22**	**0.06**	**0.22**	**0.18**	**0.21**	**0.02**	**0.01**
	stdev	*0.01*	*0.01*	*0.00*	*0.04*	*0.01*	*0.01*	*0.00*	*0.02*	*0.00*	*0.02*	*0.09*	*0.06*	*0.12*	*0.10*	*0.08*	*0.01*	*0.02*
	N	*5*	*5*	*5*	*5*	*5*	*5*	*5*	*3*	*4*	*4*	*5*	*2*	*5*	*5*	*5*	*3*	*5*
**LEVOFLOXACINE**	median	**0.05**	**0.02**	**0.03**	**0.01**	**0.88**	**0.01**	**0.01**	**0.24**	**0.00**	**0.38**	**0.01**	**0.14**	**0.01**	**0.01**	**0.01**	**0.00**	**0.00**
	stdev	*0.01*	*0.01*	*0.01*	*0.00*	*0.02*	*0.00*	*0.00*	*0.12*	*0.00*	*0.06*	*0.01*	*0.12*	*0.01*	*0.01*	*0.01*	*0.00*	*0.00*
	N	*5*	*5*	*5*	*5*	*5*	*5*	*5*	*3*	*4*	*4*	*5*	*2*	*5*	*5*	*5*	*3*	*5*
**MECILLINAM**	median	**0.01**	**0.01**	**0.01**	**0.02**	**0.00**	**0.92**	**0.03**	**0.02**	**0.01**	**0.02**	**0.01**	**0.03**	**0.01**	**0.01**	**0.01**	**0.03**	**0.03**
	stdev	*0.00*	*0.00*	*0.01*	*0.01*	*0.00*	*0.02*	*0.01*	*0.02*	*0.00*	*0.01*	*0.01*	*0.01*	*0.01*	*0.01*	*0.00*	*0.01*	*0.01*
	N	*5*	*5*	*5*	*5*	*5*	*5*	*5*	*3*	*4*	*4*	*5*	*2*	*5*	*5*	*5*	*3*	*5*
**DMSO**	median	**0.02**	**0.03**	**0.04**	**0.01**	**0.02**	**0.04**	**0.83**	**0.02**	**0.07**	**0.05**	**0.02**	**0.05**	**0.03**	**0.01**	**0.02**	**0.07**	**0.08**
	stdev	*0.01*	*0.01*	*0.01*	*0.00*	*0.01*	*0.02*	*0.05*	*0.02*	*0.04*	*0.01*	*0.02*	*0.02*	*0.02*	*0.01*	*0.01*	*0.03*	*0.02*
	N	*5*	*5*	*5*	*5*	*5*	*5*	*5*	*3*	*4*	*4*	*5*	*2*	*5*	*5*	*5*	*3*	*5*
**GyrB/ParE ATPase**	median	**0.04**	**0.03**	**0.63**	**0.01**	**0.23**	**0.02**	**0.03**	**0.90**	**0.02**	**0.30**	**0.03**	**0.27**	**0.04**	**0.02**	**0.00**	**0.03**	**0.02**
	stdev	*0.03*	*0.01*	*0.19*	*0.01*	*0.19*	*0.01*	*0.01*	*0.06*	*0.00*	*0.05*	*0.02*	*0.05*	*0.02*	*0.03*	*0.04*	*0.00*	*0.02*
	N	*3*	*3*	*3*	*3*	*3*	*3*	*3*	*3*	*2*	*3*	*3*	*2*	*3*	*3*	*3*	*3*	*3*
**MD3**	median	**0.01**	**0.03**	**0.13**	**0.01**	**0.02**	**0.17**	**0.59**	**0.06**	**0.92**	**0.04**	**0.03**	**0.09**	**0.05**	**0.02**	**0.03**	**0.71**	**0.63**
	stdev	*0.01*	*0.02*	*0.11*	*0.00*	*0.01*	*0.15*	*0.13*	*0.01*	*0.03*	*0.02*	*0.02*	*0.05*	*0.06*	*0.02*	*0.05*	*0.18*	*0.13*
	N	*4*	*4*	*4*	*4*	*4*	*4*	*4*	*2*	*4*	*3*	*4*	*2*	*4*	*4*	*4*	*2*	*4*
**NITROFURANTOIN**	median	**0.20**	**0.12**	**0.19**	**0.04**	**0.31**	**0.05**	**0.09**	**0.25**	**0.01**	**0.88**	**0.05**	**0.34**	**0.05**	**0.07**	**0.05**	**0.02**	**0.02**
	stdev	*0.08*	*0.03*	*0.12*	*0.03*	*0.04*	*0.04*	*0.03*	*0.06*	*0.01*	*0.02*	*0.01*	*0.00*	*0.02*	*0.01*	*0.03*	*0.01*	*0.01*
	N	*4*	*4*	*4*	*4*	*4*	*4*	*4*	*3*	*3*	*4*	*4*	*2*	*4*	*4*	*4*	*3*	*4*
**TRICLOSAN**	median	**0.17**	**0.28**	**0.08**	**0.15**	**0.07**	**0.09**	**0.18**	**0.03**	**0.04**	**0.14**	**0.92**	**0.04**	**0.67**	**0.64**	**0.69**	**0.02**	**0.05**
	stdev	*0.08*	*0.07*	*0.01*	*0.11*	*0.05*	*0.03*	*0.06*	*0.04*	*0.02*	*0.07*	*0.03*	*0.01*	*0.17*	*0.12*	*0.12*	*0.03*	*0.02*
	N	*5*	*5*	*5*	*5*	*5*	*5*	*5*	*3*	*4*	*4*	*5*	*2*	*5*	*5*	*5*	*3*	*5*
**TRIMETHOPRIM**	median	**0.12**	**0.07**	**0.36**	**0.06**	**0.18**	**0.10**	**0.12**	**0.35**	**0.04**	**0.49**	**0.04**	**0.89**	**0.04**	**0.04**	**0.03**	**0.04**	**0.03**
	stdev	*0.06*	*0.01*	*0.11*	*0.03*	*0.02*	*0.04*	*0.08*	*0.15*	*0.04*	*0.04*	*0.02*	*0.02*	*0.03*	*0.01*	*0.03*	*0.04*	*0.04*
	N	*2*	*2*	*2*	*2*	*2*	*2*	*2*	*2*	*2*	*2*	*2*	*2*	*2*	*2*	*2*	*2*	*2*
**1a**	median	**0.27**	**0.26**	**0.07**	**0.21**	**0.08**	**0.06**	**0.16**	**0.05**	**0.02**	**0.14**	**0.71**	**0.06**	**0.92**	**0.79**	**0.82**	**0.02**	**0.03**
	stdev	*0.12*	*0.08*	*0.02*	*0.12*	*0.06*	*0.02*	*0.08*	*0.04*	*0.02*	*0.06*	*0.07*	*0.01*	*0.03*	*0.06*	*0.12*	*0.03*	*0.01*
	N	*5*	*5*	*5*	*5*	*5*	*5*	*5*	*3*	*4*	*4*	*5*	*2*	*5*	*5*	*5*	*3*	*5*
**1b**	median	**0.30**	**0.24**	**0.07**	**0.18**	**0.08**	**0.05**	**0.09**	**0.04**	**0.02**	**0.14**	**0.72**	**0.05**	**0.69**	**0.92**	**0.74**	**0.02**	**0.02**
	stdev	*0.15*	*0.08*	*0.01*	*0.12*	*0.05*	*0.02*	*0.06*	*0.04*	*0.01*	*0.06*	*0.12*	*0.01*	*0.09*	*0.03*	*0.12*	*0.02*	*0.01*
	N	*5*	*5*	*5*	*5*	*5*	*5*	*5*	*3*	*4*	*4*	*5*	*2*	*5*	*5*	*5*	*3*	*5*
**1h**	median	**0.29**	**0.25**	**0.07**	**0.19**	**0.07**	**0.06**	**0.13**	**0.03**	**0.02**	**0.15**	**0.73**	**0.04**	**0.80**	**0.75**	**0.92**	**0.01**	**0.03**
	stdev	*0.12*	*0.08*	*0.01*	*0.10*	*0.04*	*0.02*	*0.07*	*0.04*	*0.01*	*0.07*	*0.03*	*0.02*	*0.06*	*0.08*	*0.01*	*0.02*	*0.01*
	N	*5*	*5*	*5*	*5*	*5*	*5*	*5*	*3*	*4*	*4*	*5*	*2*	*5*	*5*	*5*	*3*	*5*
**1j**	median	**0.02**	**0.02**	**0.08**	**0.01**	**0.01**	**0.36**	**0.45**	**0.08**	**0.77**	**0.06**	**0.03**	**0.10**	**0.05**	**0.02**	**0.04**	**0.92**	**0.75**
	stdev	*0.01*	*0.03*	*0.16*	*0.00*	*0.01*	*0.06*	*0.11*	*0.04*	*0.02*	*0.02*	*0.01*	*0.07*	*0.01*	*0.01*	*0.01*	*0.02*	*0.05*
	N	*3*	*3*	*3*	*3*	*3*	*3*	*3*	*3*	*2*	*3*	*3*	*2*	*3*	*3*	*3*	*3*	*3*
**1o**	median	**0.01**	**0.03**	**0.17**	**0.01**	**0.02**	**0.23**	**0.51**	**0.08**	**0.74**	**0.05**	**0.04**	**0.09**	**0.05**	**0.03**	**0.03**	**0.87**	**0.91**
	stdev	*0.01*	*0.04*	*0.12*	*0.02*	*0.01*	*0.14*	*0.08*	*0.04*	*0.19*	*0.02*	*0.02*	*0.07*	*0.03*	*0.01*	*0.02*	*0.18*	*0.04*
	N	*5*	*5*	*5*	*5*	*5*	*5*	*5*	*3*	*4*	*4*	*5*	*2*	*5*	*5*	*5*	*3*	*5*

Table-S3 -Identification of MoA for new antibacterial compound classes

Similarity score for compound effect on E. coli ΔTolC derived using Random Forest based classification in 2-5 experiment series, each 2 independent experiments. Column 1-7. Standard set of reference compounds, median value. Each of the following columns is for the similarity score generated with a slightly different reference set where the indicated test

compound has replaced the compound with the closest profile in the reference set. Only the similarity score towards the new reference compound is displayed.
